# Chitosan in Non-Viral Gene Delivery: Role of Structure, Characterization Methods, and Insights in Cancer and Rare Diseases Therapies

**DOI:** 10.3390/polym10040444

**Published:** 2018-04-15

**Authors:** Beatriz Santos-Carballal, Elena Fernández Fernández, Francisco M. Goycoolea

**Affiliations:** 1 ChiPro GmbH, Anne-Conway-Street 1, 28359 Bremen, Germany; bcarballal@chipro.de; 2 Lung Biology Group, Department Clinical Microbiology, RCSI, Education and Research Centre, Beaumont Hospital, Dublin 9, Ireland; elenaffernandez@rcsi.ie; 3 School of Food Science and Nutrition, University of Leeds, Leeds LS2 9JT, UK

**Keywords:** gene delivery, non-viral vectors, chitosan structure, pDNA, siRNA

## Abstract

Non-viral gene delivery vectors have lagged far behind viral ones in the current pipeline of clinical trials of gene therapy nanomedicines. Even when non-viral nanovectors pose less safety risks than do viruses, their efficacy is much lower. Since the early studies to deliver pDNA, chitosan has been regarded as a highly attractive biopolymer to deliver nucleic acids intracellularly and induce a transgenic response resulting in either upregulation of protein expression (for pDNA, mRNA) or its downregulation (for siRNA or microRNA). This is explained as the consequence of a multi-step process involving condensation of nucleic acids, protection against degradation, stabilization in physiological conditions, cellular internalization, release from the endolysosome (“proton sponge” effect), unpacking and enabling the trafficking of pDNA to the nucleus or the siRNA to the RNA interference silencing complex (RISC). Given the multiple steps and complexity involved in the gene transfection process, there is a dearth of understanding of the role of chitosan’s structural features (*M*_w_ and degree of acetylation, DA%) on each step that dictates the net transfection efficiency and its kinetics. The use of fully characterized chitosan samples along with the utilization of complementary biophysical and biological techniques is key to bridging this gap of knowledge and identifying the optimal chitosans for delivering a specific gene. Other aspects such as cell type and administration route are also at play. At the same time, the role of chitosan structural features on the morphology, size and surface composition of synthetic virus-like particles has barely been addressed. The ongoing revolution brought about by the recent discovery of CRISPR-Cas9 technology will undoubtedly be a game changer in this field in the short term. In the field of rare diseases, gene therapy is perhaps where the greatest potential lies and we anticipate that chitosans will be key players in the translation of research to the clinic.

## 1. Introduction

Modern understanding of health is based on the concept of regulation of metabolism by a complex network of molecular-based communication mechanisms known as cell signaling that governs basic cellular activities and coordinates cell responses so that they can act in concert. Cells in the body perform their life cycle functions partially by genetic programming, but also by responding to molecular signals generated within the cell. These networks respond to, are controlled by, and can be disrupted by processes that take place on the electrical, molecular, macromolecular and supramolecular scales [1]. These are also the domains of nanoscience and nanotechnology. Nanomedicine has emerged as a recent multidisciplinary field in which the manipulation of matter at such scales is used for diagnostics and therapy to tackle the current most important challenges in health under innovative, affordable and more effective approaches. 

Even when extensive research is conducted focusing on advanced nanobiomaterials to be used in biomedicine or biotechnology, that their design and biophysical properties can be finely tuned, and their effectiveness in vitro or in vivo as drug and gene carriers has been demonstrated, sound fundamental understanding of the mechanistic aspects at the molecular and cell level is still lacking. Moreover, in the case of biopolymer-based biomaterials, there is a general lack of studies that enable establishing robust structure-function relationships and, hence, a more rational design of innovative biomaterials. This is particularly relevant for the development of new and more effective nanomedicines to treat cancer, autoimmune diseases, viral and antibiotic-resistant bacterial infections, and genetic rare diseases. At the same time, gene therapy has started to deliver results in clinical trials, and it is considered to be at a booming stage. Children suffering devastating diseases have seen their lives transformed. In the last two decades, gene therapy has caught significant attention as a potential method for treating genetic disorders such as cystic fibrosis [2], Parkinson’s disease [3,4] as well as an alternative method for treating cancer [5]. The discovery of CRISPR (clustered regularly interspaced short palindromic repeats) sequences in DNA as part of the immune system of bacteria and archae, and the subsequent rise of CRISPR-Cas9 technology that has enabled the genome editing of plant and animal cells and to target disease-related genes in patients, has brought gene therapy to a brand new light. Indeed, an ongoing revolution in the therapeutic paradigms for many diseases, particularly genetic diseases, is currently underway, and we have only started to see the “tip of the iceberg” [6]. 

Another driver that accelerated the recent progress in gene therapy over the past years is the basic research into novel vectors for gene delivery. The use of genetically engineered recombinant viruses, namely adenoviruses and adeno-associated viruses, that carry the therapeutic gene payload in their viral capsid, thus protecting it from enzymatic degradation, has been the most pursued route to bring gene therapy from the laboratory bench to the bedside. This includes recent advances in vivo delivery of the Cas9 therapeutics [7]. However, this approach poses serious shortcomings and challenges due to the widespread human immunity against viruses, off-target genomic damage and their small packing size. Hence, the search for non-viral vectors has also geared substantial research in this field [8]. In the case of CRISPR-Cas9 technology, a recent paper has documented the use of gold nanoparticles conjugated with DNA and synthetic polymer as an advanced co-delivery strategy for Cas9 ribonucleoprotein and the donor DNA payload [9].

For more than two decades, chitosan has been a highly researched non-viral gene delivery biopolymer. However, it is still not fully understood what type of chitosan works best for the different type of therapeutic genes (e.g., pDNA, siRNA, miRNA, etc.) or the role of their conformations (e.g., single versus double stranded), and the shape and size of the complexes (also regarded here indistinctly as “polyplexes” or “nanocomplexes”). The breath of this review is on the utilization of chitosan as a non-viral gene delivery vector. To put this in the current context of knowledge, we give an overview of the current progress on viral and non-viral vectors for gene delivery. We review the current understanding on the role of chitosan’s molecular structure and the efficacy to deliver different type of polynucleotides to mammalian cells. The focus of this paper circumscribes only to unmodified chitosans. A separate section addresses the major methods available to characterize the biophysical properties of non-viral gene delivery systems. This is followed by another two sections in which we focus on the advances on plasmid DNA and silencing RNA delivery and the current progress on gene therapy in the treatment of rare diseases of which we review the major types and their specific challenges. Even when most pre-clinical research has been geared towards viral vectors, chitosan-based non-viral systems have started to offer promising results in vitro (e.g., cystic fibrosis gene therapy). 

Finally, we draw a few conclusions on the current gaps of the current knowledge, unmet challenges and future perspectives of chitosan as key players in the potential future translation of the wealth of in vitro and pre-clinical proof of concept research studies. Even though excellent comprehensive reviews that address non-viral gene delivery, including chitosan and its chemical derivatives, and combinatorial therapies, have recently been published [10,11,12,13,14,15,16,17], we have attempted in our review to bring together the current status of scattered knowledge related to chitosan non-viral gene delivery and its potential to replace viral vectors, particularly for rare diseases therapy.

## 2. Gene Therapy: Viral and Non-Viral Vectors in Gene Delivery 

The main goal of gene therapy is to introduce new genetic material into targeted cells in the body. This approach provides several advantages compared to conventional protein therapy. Through the introduction of exogenous nucleic acids into a specific cell, it is possible to control and modulate the genomic expression. The direct transfer of a specific gene into a patient will lead to in vivo production of proteins in the target cells as “mini-bioreactors” [18]. Ideally, this process will occur at more physiological conditions than those achieved by conventional administration of therapeutic proteins [19,20]. Therefore, the simple approach of using therapeutic genes as a “pro-drug” to treat a patient may result in an alternative way to overcome the drawbacks associated with the use of recombinant proteins. The effectiveness of gene therapy relies on several feats, namely protection of the nucleotides from premature degradation in the extracellular environment, targeting of specific cells, and delivery of sufficient amounts of genetic material to produce a therapeutic effect [21]. Therefore, the major challenge related to gene therapy is the development of a non-toxic, non-immunogenic and effective intracellular delivery system. To find suitable vehicles for the efficient delivery of therapeutic oligo- and polynucleotides at the expense of minimal toxicity and immunogenicity, both viral and non-viral vectors have been developed.

**Figure 1:**
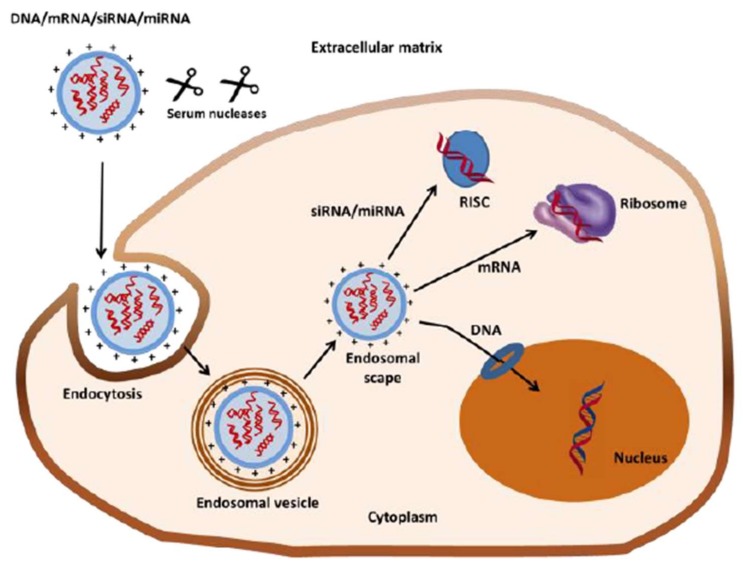
Barriers to successful in vivo delivery of nucleic acids.

After the administration of genetic material, a sequence of biochemical and physical barriers must be overcome. These are shown schematically in Figure 1, and are described as follows:

- The existence of serum nucleases in the extracellular environment results in rapid degradation of genetic material on intravenous, mucosal and intramuscular administration.
- The entry of nucleic acids (e.g., DNA, mRNA, and siRNA/miRNA) into the cell is mainly restricted to the endocytotic pathway, but the association of nucleotides with the cell surface is very low as a consequence of the high negative charge of the polynucleotides and the proteoglycans present in the cell membrane [22].
- Once internalized by the specific cells, the genetic material has to escape from the endosomal vesicle, where the low pH and enzymes present can lead to its degradation [23,24].
- DNA has to diffuse in the cytosol to get into the nucleus; this diffusion process is size-dependent and DNA larger than 3000 base pairs present highly reduced mobility [23,24].
- siRNA and miRNA must be loaded into the RNA-induced silencing complex (RISC), whereas mRNA must bind to the translational machinery [25].

To improve the efficacy of the deliver machinery of genes across these barriers, the nucleic acids can be inserted into vehicles for gene delivery to assist the transfer of exogenous genes to the specific cells. These vehicles can be divided in two groups: viral vectors and non-viral vectors.

### 2.1. Viral Vectors in Gene Delivery

The approach used in viral gene delivery is based on the ability of vectors to infect cells. This involves the use of genetically engineered recombinant viruses, adenoviruses and adeno-associated viruses that carry the therapeutic gene in their viral capsid, thus protecting it from enzymatic degradation [26]. Viral vectors have been developed based on a wide range of viruses and typically include strong promoters that achieve a high level of heterologous gene expression. Over the past two decades, more than 1800 clinical trials have been completed, are ongoing or have been approved worldwide in more than 30 countries [27]. However, these trials have only yielded five products globally. These products include Gendicine, Oncorine, Rexin G, Neovasculgen, and Glybera. In Europe, Glybera was approved for the treatment of patients with a familial lipoprotein lipase deficiency (LPLD) and, after some years, was discontinued; other formulations for cancer, myocardial ischemia, Duchenne muscular dystrophy, and painful diabetic neuropathy are currently in Phase III of clinical trials [28,29]. The large majority (~90%) of clinical trials utilized viral vectors, such as adenoviruses, adeno-associated viruses (AAVs), lentiviruses, or retroviruses. The low success rate of approved products underscores the many challenges that the utilization of viral vectors entails, as discussed below.

Viruses are biological particles that have evolved to transfer genetic information from one cell to another. The simplest viral particles consist of a protein coat that surrounds a strand of nucleic acid. They vary in the composition of their capside envelope, as well as in size and morphology. Hence, they occur as nanospheres of size ranging from ~20 nm (e.g., adeno-associated virus) to ~100 nm (e.g., adenovirus); short (~300 nm) nanorods (e.g., tobacco mosaic virus) and long (~700 nm) rods (e.g., Stygiolobus virus); worm-like particles (~1 μm; e.g., Ebola virus); and other shapes such as ellipsoid-, and cubic-shaped structures (e.g., *Acidianus convivator*). Moreover, viral genomes are diverse in size, structure, and nucleotide composition. They can be linear or circular chains of dsDNA, ssDNA, dsRNA or ssRNA. Given this great diversity, viruses offer a vast source of bioinspiration for bottom-up nanobiotechnology biomimetic approaches to conceiving synthetic novel vectors for gene delivery. This is particularly relevant, as the use of viral vectors in therapy raises significant safety issues, such as potential immunogenicity and reversion to pathogenicity of the vector [26]. Moreover, undesired mutations through the integration of their DNA into the genome of the introduced cells may lead to insertional mutagenesis and oncogenesis [19,30]. Other shortcomings include: limited DNA packaging capacity, complex production processes, broad tropism, and cytotoxicity. This has encouraged the development of non-viral vectors with better biosafety profiles and potential to address many of the limiting issues of viral vectors, thanks to the advances in materials science, nucleic acids chemistry and nanobiotechnology [31]. The most widely studied non-viral vectors include: cationic lipids, cell penetrating peptides, and cationic macromolecules. The major aspects of these systems are discussed in the next subsection.

### 2.2. Non-Viral Vectors in Gene Delivery and the “Proton Sponge” Hypothesis

The development of non-viral vectors aims to reach at least the same level of gene expression and specificity obtained when using viral vectors. The advantages of the use of non-viral vectors are related to their low cost and ease of production, their reduced immunogenicity and immunotoxicity and, therefore, greater bio-safety, in comparison to viral-mediated gene therapy. Non-viral vectors can be produced in a large scale, they are more flexible for optimization and control of the formulation and they are able to deliver large DNA sequences [23,32]. Non-viral vectors comprise naked nucleic acids or more complex systems based on the use of cationic molecules such as lipids, cell penetrating peptides or polycationic macromolecules. The nature of interaction between these non-viral vectors and the opposite negatively charged nucleic acids is mainly electrostatic, involving indeed, to transfer the genes effectively, it is desired a delivery system that carry a net positive charge to facilitate the interaction with the negatively charged cell membrane. Afterwards, it is hypothesized that the internalization into the cytoplasm will occur via endocytosis [33]. In this section, we briefly present the main type of used non-viral vectors to give the broad context of chitosan as one of the major non-viral gene delivery systems. 

#### 2.2.1. Cationic Lipids

Lipid-based vectors are among the most widely used non-viral gene carriers. Cationic lipids present amino groups to interact with nucleotides and hydrophobic groups constituted by fatty acids. The hydrophobic moieties contribute to the formation of bilayer vesicles in an aqueous medium. The first report on the use of lipid carriers is from 1987 with the introduction of *N*-[1-(2,3,-dioleyloxy)propyl]-*N*,*N*,*N*-trimethylamonium [34]. Since then, several cationic lipids have been synthesized and studied for gene delivery [35,36,37,38,39,40]. Lipocomplexes (also termed “lipoplexes”) are promising candidates for in vitro and in vivo gene delivery [37,41]. However, these systems present some limitations including poor stability and rapid clearance, as well as the generation of an inflammatory response and relatively high cytotoxicity [26].

#### 2.2.2. Cell Penetrating Peptides (CPPs)

CPPs are peptides containing domains of less than 20 amino acids and are characterized to interact specifically with receptors in the cell membrane and can transport molecules across it. They have gained popularity as non-viral transmembrane delivery vectors. CPPs are employed to enhance extracellular and intracellular internalization of relevant biomolecules including nucleotides. Gene delivery mediated by CPPs is classified in covalently bound and electrostatically bound. Either way, the CPPs are used to promote the delivery of their associated drugs and drug carriers into cells facilitated by an active transport mechanism. The most commonly used CPP is TAT peptide (TATp), derived from the transcriptional activator protein encoded by human immunodeficiency virus type 1 (HIV-1). TATp has been used for intracellular delivery in a range of cell types both in vitro and in vivo [42,43,44,45]. CPPs have recently been used to deliver Cas9 protein-RNA complex to enable rapid and timed editing with potential in human gene therapy [46].

#### 2.2.3. Cationic Macromolecules

Cationic macromolecules have emerged as an alternative type of carriers for gene therapy. Macromolecules with functional groups able to be protonated at physiological pH, thus bearing positive charges, can be complexed with the negatively charged phosphates groups from nucleotides in nucleic acids [47,48,49]. The resulting systems are regarded as “polyplexes” or simply “complexes” and are self-assembled systems that exert their properties depending on the (+/−) charge ratio used. 

The compaction of DNA by multivalent cations was studied by Matulis et al. using isothermal titration calorimetry (ITC) [50]. The model proposed by the authors consists of a two-stage process: (1) the cation binds to DNA through non-specific electrostatic forces, resulting in neutralization of the charges of the nucleotides, a decrease in charge repulsion and therefore an increase in the flexibility of the chains; and (2) after reaching a critical ligand concentration, DNA-DNA interactions occur and they condense into self-assembled systems, an entropically driven process [50]. In the specific case of polymeric cations (e.g., chitosan and PEI), it has been observed that the compaction of DNA occurs in a less tight manner, producing larger complexes than those formed with multivalent cations [51]. The condensation of smaller nucleic acids like siRNA by cationic polymers has been studied; it is believed that this process comprises the presence of many siRNA molecules in the formation of nanoparticles through interparticle assembly [52].

Among the main advantages of the use of cationic macromolecules are their ease for functionalization and possibility of binding specific targeting moiety, their higher stability compared to lipoplexes and lower cytotoxicity. Cationic macromolecules can be synthetic such as poly(l-lysine), polyethyleneimine and dendrimers or natural such as poly(d,l-lactic acid) and chitosan [23,26,32]. Despite the extensive reported progress on non-viral gene vectors, these systems still present deficient expression of their transgenes when transfecting mammalian cells as compared to viral systems [53]. This has demanded many research efforts on developing suitable non-viral vectors able to protect the nucleic acids against degradation, achieve specific cell targeting, promote cellular uptake, and induce minimal cytotoxicity and immunogenic rates.

**Figure 2:**
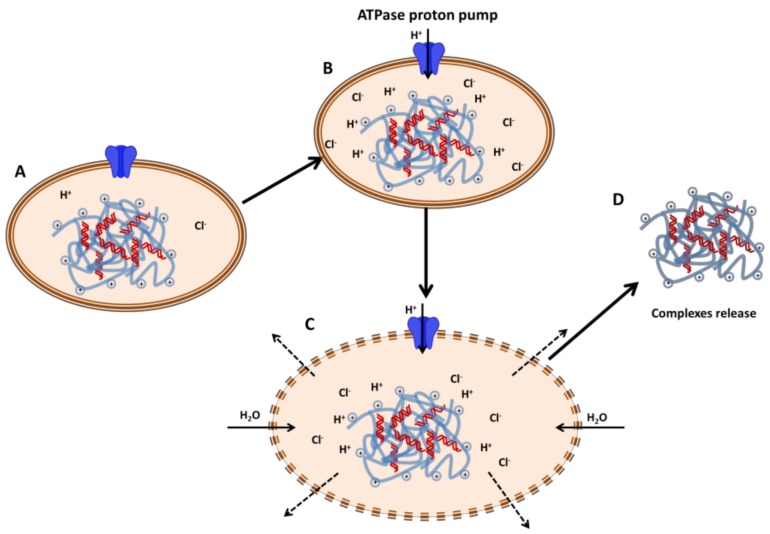
Schematic representation of the “proton sponge” hypothesis in which the endosomes containing the complexes with protonable polymers (**A**) evolve to late endosomes where protons are introduced by ATPase proton pumps, producing protonation of the polymer and a reduction in the pH (**B**); subsequently, chloride ions will be introduced in a non-active way causing a water inflow due to the osmotic pressure (**C**). Swelling of the endosomes leads to their rupture and finally release of their content into the cytoplasm (**D**).

Among the cationic polymers used for gene transfection, it has been observed that polymers containing amine groups with pKas around physiological pH lead to the best transgene expression. It is hypothesized that these systems exhibit “proton sponge” potential [54]. The “proton sponge” hypothesis has been described as the followed route to induce endosomal disruption and prevent nucleic acids from lysosomal degradation. Figure 2 describes the process after endocytosis of the complexes. Throughout the evolution of the endosomes, protons are translocated by ATPase proton pumps from the cytoplasm into the endosomes. This will cause a reduction in the pH of the endosomal compartments and the protonation of the cationic polymers with “proton sponge” potential. Therefore, more protons will be pumped in and chloride ions will passively accumulate into the endosomes. The increase in the ionic concentration inside the endosomes will cause water inflow, swelling and rupture of the endosomes and release of their content into the cytoplasm [33,54].

Polymers like polyethyleneimine and dendrimers containing protonable secondary or tertiary amine groups possess good rates of transfection efficiency both in vitro and in vivo [55,56,57,58,59]. On the other hand, chitosans are reported to have low proton sponge capacity [60,61]. However, Richard et al. have found that CS (*M*_w_~8 kDa and DA = 8%) presents similar capability as polyethyleneimine to induce proton sponge effect, and, therefore, to mediate endosomal escape. One of the main considerations has been that previous studies were carried out using mass concentration of chitosan instead of concentration on N-glucosamine units, leading to underestimation of the potential of chitosan to produce effective endosomal release [62]. Even when the proton sponge hypothesis offers a general explanation to the endolysosomal escape of polycationic non-viral vectors during intracellular trafficking, it is not the only mechanism at play that limits the rate of transit of the nucleic acid payload or the transfection efficiency [63]. 

## 3. Chitosan as a Non-Viral Gene Delivery Vector 

As described above, polymers with cationic characteristics such as chitosan present enormous potential as gene delivery carriers. The primary amines in the chitosan backbone are protonated at slightly acidic pH, resulting in positive charges available to interact with nucleic acids via electrostatic forces. Mumper et al., have reported the use of chitosan as a non-viral gene delivery system for plasmid transfection for first time in 1995 [64]. In 1998, the use of chitosan in in vivo applications and its potential for the delivery of nucleic acids in mucosal epithelia (e.g., nose and lung) was documented [47]. There are several subsequent studies on the potential use of chitosan and its derivatives for the delivery of DNA [65,66]. From 2006 onwards, chitosan has been also used for condensing short interfering RNA (siRNA) [67,68,69,70]. More recently, chitosan–miRNA complexes have been investigated to target cystic fibrosis cells [71]. 

Thus far, only few studies have systematically investigated how the structure of chitosan, specifically the degree of acetylation (DA), and degree of polymerization (DP), affects the biophysical characteristics and biological functionality of chitosan-based systems. Indeed, attempts have been made to establish a relationship between the DP and DA, the salt form and pH, on the efficiency of transfection with plasmid DNA in vitro [72,73,74,75,76] and to determine the intracellular trafficking routes underlying their mode of action [77]. An ideal balance between the strength of the interaction between chitosan and plasmid DNA and the dissolution of the complex within the cell (thus conferring optimal transfection efficiency) can be achieved using chitosan molecules with specific *M*_w_ and DA [78]. The biophysical properties of chitosan–siRNA complexes and their capacity for transfection have been investigated in detail [79]. Further studies suggest that optimal properties include the use of chitosans of low molecular weight and high DA, and complexes of small particle size (~100 nm) and a moderate positive surface zeta potential along with a high (N/P) charge ratio [80]. Efforts to elucidate the role of the molecular structure of chitosan (*M*_w_ and DA) in terms of the major barriers, including internalization, endolysosomal escape, unpacking, and nuclear entry, that limit the transfection efficiency of chitosan–pDNA polyplexes, have been carried out in HEK293 cells [77]. This cell line is very popular for being easy to culture and to accept foreign DNA. The kinetics of the polyplex decondensation in relation to lysosomal sequestration and escape on chitosan–DNA systems have been suggested to be critically dependent on chitosan’s structure. A conclusion that emerged from these studies was that chitosans that promote relatively stable polyplexes, but not too stable (e.g., low DA and high *M*_w_), are optimal (DA~20% *M*_w_ 40 kDa or DA~8% *M*_w_ 10 kDa). Our own results on chitosan–microRNA polyplexes are consistent with this view, as we have evidenced that chitosans of intermediate affinity to bind miRNA (DA~20% *M*_w_ 26 kDa) have the greatest transfection efficiency in MCF-7 cells [81]. Despite the experimental evidence available, there is not yet a firm theory explaining how these factors contribute to the observed transfection efficiency in DNA and RNA systems.

## 4. Supramolecular Chitosan-Based Nanostructures for Gene Delivery

**Figure 3:**
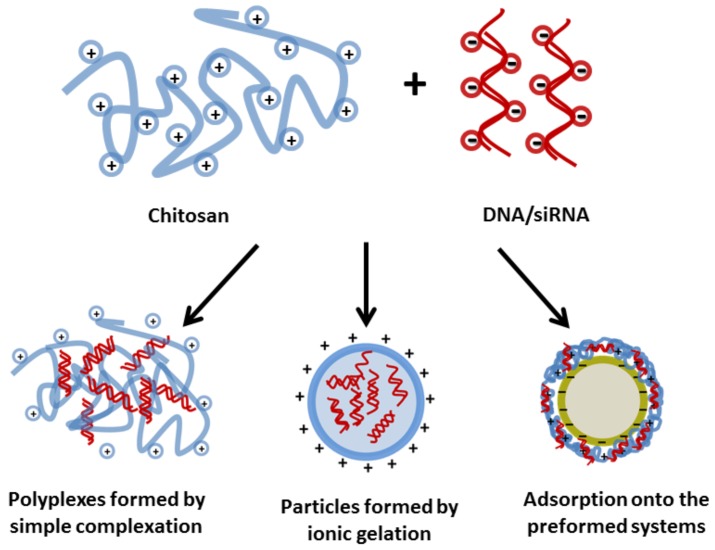
Preparation of chitosan-based DNA/siRNA nanoparticles following different strategies.

Chitosan based systems can be prepared following three main techniques: simple complexation, ionic gelation using crosslinkers and adsorption of DNA/siRNA onto the surface of preformed chitosan nanoparticles (Figure 3) [10,69]. 

The formation of self-assembled complexes with polynucleotides by direct mixing of the components in water is the simplest method. As already mentioned, the formation of such complexes is driven by electrostatic forces in aqueous solution. Despite the simplicity of the method, there are some issues that must be carefully adjusted concerning the mixing conditions, the ratio of charges used, as well as the characteristics of the chitosans. It is reported in a patent by Buschmann et al. that efficient transfecting complexes are formulated by adding chitosan over the nucleotides, pipetting up and down, tapping the tube gently and further incubating for 30 min [82].

The preparation of the delivery system using ionic gelation is based on the ability of chitosan to undergo a sol-gel transition due to the ionic interaction with a polyanion [83,84]. The addition of a third component (e.g., pentasodium tripolyphosphate, TPP) is reported to reduce the size of the particles and to increase the stability of complexes during their incubation in biological fluids [67,84,85]. Chitosan-crosslinked nanoparticles are suitable for the simultaneous encapsulation and sustained release of DNA molecules. Recently, Rafiee et al. showed the preparation of hydrogel nanoparticles with encapsulated plasmid, the system was prepared by simple complexation of chitosan and DNA and in a second step the addition of alginate to protect DNA while forming the hydrogel [86].

Adsorption of DNA on the surface of nanoparticles has been reported using preformed chitosan–TPP–hyaluronic acid nanoparticles. The particles were prepared by ionic gelation and in a further step the plasmid was added, showing efficient transfecting in ocular gene therapy [87,88].

### 4.1. Effect of the Degree of Acetylation

The degree of acetylation of chitosan is directly related with the density of positive charges along the chitosan chain. Chitosans of low DAs generate high density of positive charges, meaning a greater number of sites for nucleotides binding and improved capacity to interact with the cellular membrane surface and hence, to favor the uptake [89]. The average particle size diameter of complexes formed with either pDNA or siRNA is also known to be affected by the DA; as a general trend is found an increase in size with an increment of the acetylated units on the chitosan chain [10,73,90].

**Figure 4:**
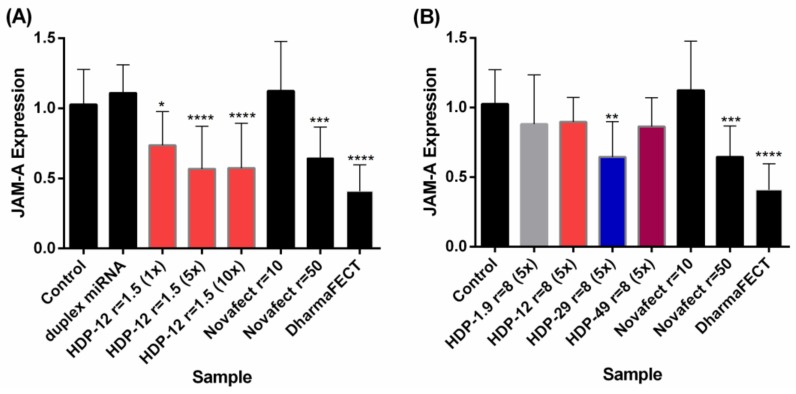
Transfection efficiency expressed as downregulation of JAM-A mRNA in MCF-7 cells: (**A**) complexes containing CS HDP-12 at (N/P) charge ratio = 1.5; and (**B**) complexes containing CS HDP-1.9, HDP-12, HDP-29 and HDP-49 at (N/P) charge ratio = 8. Duplex miRNA (dose 1× = 0.05 nmol/well), DharmaFECT (5 µL/well) and Novafect O 25 were used as controls. Data represent mean values (± SD) of three independent biological experiments and three technical replicates. Statistical comparisons were between each treatment and the control of untreated cells using non-parametric Kruskal–Wallis test (* *p* < 0.1; **; *p* < 0.01***; *p* < 0.001; **** *p* < 0.0001). Source Santos-Carballal et al. Scientific Reports 5, Article number: 13567 (2015) doi:10.1038/srep13567 [81], licensed under a Creative Commons Attribution 4.0 International License.

It has been confirmed that the chitosan’s DA affects pDNA binding, release and gene transfection efficiency in vitro and in vivo [91]. Koping-Hoggard et al. reported that the DA must be lower than 35% to obtain stable complexes with DNA that transfect HEK293 cells [72]. An increase in the DA decreases the stability of the particles in presence of serum proteins and components from the medium, thus decreasing the transfection efficiency in HEK293, HeLa and SW756 cells [91]. Liu et al. studied the influence of the structural properties on chitosan–siRNA nanoparticle and its influence on gene silencing in H1299 human lung carcinoma cells. The highest gene silencing efficiency was achieved under specific characteristics: DA = 16% and high molecular weight using chitosan–siRNA nanoparticles at N/P 150 [48]. Our own work on delivering hsa-miRNA-145 to MCF-7 breast cancer cells revealed that the greatest transfection efficiency, measured in terms of the downregulation of the target gene, was observed for polyplexes of chitosan with DA 29%, when compared with a series of DA between 1.6% and 49% (*M*_w_ ~18–26 kDa), as shown in Figure 4 [81]. When we measured the association and dissociation affinity constants for the binding of the series of chitosans with the hsa-miRNA-145, we confirmed that the chitosan with DA 29% had an intermediate binding affinity [81,92] (see Section 5, Surface Plasmon Resonance). Recently, an exhaustive study on the uptake of chitosan–siRNA polyplexes and their transfection efficiency in vitro and in vivo was reported by Alameh et al. Their results show a predominant effect of chitosan’s DA on controlling the charge density of the complexes, and the most successful in vitro knockdown rates were obtained with chitosan (DA 28% *M*_w_ 10 kDa). In agreement with previous reports, they also experienced that the degree of polymerization and the N/P ratio had a minor effect on the knockdown efficiency [93].

The role of the pattern of acetylation (PA) that defines the distribution of *N*-acetyl-d-glucosamine residues along blocks of charged poly-d-glucosamine in the chitosan chains, on the specific interactions between chitosan and DNA or RNA is widely unknown. Ongoing efforts in our laboratories are being made in this direction and the first enzymatically patterned chitosans have started to become available for the studies in this direction. 

### 4.2. Effect of the Molecular Weight

The length of the chitosan chain has a prominent influence on the particle size, stability, dissociation of the complexes after internalization in the cytoplasm and therefore an impact on the final transfection efficiency [94]. In general, it has been found that the size of the complexes decreases as the molecular weight is reduced [73]. However, the complexes formed between chitosan with low *M*_w_ (10–17 kDa, DA = 12%) and pDNA tend to increase in size [73]. Our own studies with polyplexes of chitosan of very low *M*_w_ chitosan (~1.2–2.0 kDa, DA~1.6–67%) and microRNA revealed that, even when the overall average size and polydispersity and zeta potential of the polyplexes did not differ much from those of the systems obtained with chitosans of low *M*_w_ (~18–26 kDa, DA~1.9% to 49%), they aggregated in RPMI minimal cell culture medium [81]. Sato et al. showed that chitosans with high *M*_w_ (>100 kDa; DA = 8%) are poorly effective in transfecting DNA, whereas chitosans with low *M*_w_ (~15 and ~52 kDa, DA = 20% and 6%, respectively) largely promote pDNA luciferase expression in several cell lines (namely A549, B16 and HeLa cells) [94], in line with our results for microRNA transfection of MCF-7 breast cancer cells [81]. Recently, Bordi et al. have compared the transfection efficiency of chitosan oligomers and chitosan of *M*_w_ 50 kDa (DA = 34%) complexed with pDNA in various cell lines. In all tested cells, the chitosan of *M*_w_ 50 kDa performed better than the oligomers, thus providing evidence of higher protection of pDNA and stability of these complexes conferred by chitosan [95]. In the Laboratory of Maria J. Alonso, where nanoparticles of chitosan by ionotropic gelation with TPP were discovered, they formulated pDNA and short dsDNA oligonucleotides into chitosan/TPP prepared with chitosan of varying *M*_w_. Low *M*_w_ chitosan (10 kDa) provided more compact nanocarriers (~100 nm) compared to high *M*_w_ chitosan (125 kDa) because of the lower viscosity of the former polymer dispersion, in consonance with previous studies [96]. Importantly, the efficiency of transfection also seemed highly dependent on the chitosan *M*_w_, with low *M*_w_ chitosan–TPP NPs exhibiting superior gene transfer in vitro. In addition, low *M*_w_ chitosan–TPP particle displayed a marked transgene expression following intratracheal administration in mice. This was comparable with the corresponding *M*_w_ chitosan–DNA polyplexes prepared in the absence of TPP though [97].

Katas and Alpar (2006) have shown that siRNA molecules are efficiently condensed and protected by high *M*_w_ chitosan (*M*_w_ = 110 and 270 kDa and DA = 14%) [67]. Furthermore, the molecular weight will influence the capacity of chitosan to entangle siRNA and the final size of the chitosan–siRNA complexes, which seems to be yet another aspect at play in cellular uptake [48,67]. It has been suggested that only chitosan molecules that were 5–10 times the length of siRNA (*M*_w_ of 13.36 kDa) could form suitable nanocomplexes. Thus, the chitosan optimal *M*_w_ recommended to obtain nanocomplexes with siRNA is 64.8–170 kDa [66]. Evidence is consistent with the notion that the *M*_w_ of chitosan can be used to tweak the average particle size of the obtained complexes [10]. It has also been found that the *M*_w_ of chitosan has an influence on the morphology. Spherical or elongated and irregular nanoparticles were formed with chitosans of *M*_w_ 44 (DA 14%) or 143 kDa (DA 22%), respectively, as evidenced by TEM and SAXS [98]. The influence of the morphology of polyplexes on the cellular uptake, intracellular trafficking and transfection efficiency, has only been recently examined on synthetic brush polymers of methacrylamide-oligolysine–pDNA (pCMV-Luc2) polyplexes [99]. In the case of chitosan-based systems, it is known that the structure (*M*_w_ and DA) determines the morphology, but how this dictates biological activity, is a so far widely neglected aspect. 

Recently, it has been seen that increased chain length increased biological performance, and that a certain *M*_w_ threshold of 10 kDa (~60−70 monomers) is required to achieve eGFP knockdown efficiency. Particles formed with chitosan of *M*_w_ below this value (e.g., 5 kDa) are found to have a reduced stability in serum and therefore low transfection rates [93]. Most likely, the interactions governing these types of systems are highly cooperative in nature. Hence, for each type of nucleic acid, there must exist a minimum cooperative length between chitosan and the nucleic acid that favors the interaction and influences the overall molecular architecture of the assembled structures. From the practical standpoint, it is very important to carefully select the optimal *M*_w_ and DA of chitosan to obtain desirable physicochemical properties and achieve proper transfection rates. The size of the particles will be determined by the molecular mass of chitosan and this will influence the stability of chitosan–nucleic acid complexes and their biological activity in vitro, namely: cellular uptake, dissociation and processing of DNA within the cells. 

### 4.3. Effect of the N/P Charge Ratio

The N/P charge ratio is defined as the ratio between the protonated amines from the chitosan and the negatively charged phosphate groups from nucleic acid, also used indistinctly in this review as the (+/−) charge ratio. The N/P charge ratio can assume values from 0 to 1, for negatively charged complexes, or values higher than 1 that correspond to positively charged complexes. Complexes with stoichiometric equivalent amount of charges are not desired since these systems are unstable and agglomerate and precipitate due to the absence of repulsive forces [49]. The positive surface net charge of the complexes influences directly the interaction with the negatively charged glycosaminoglycan molecules from the glycocalyx of the cell membrane [69]. 

N/P charge ratios below 1 imply defect of chitosan and surplus of the nucleic acid in the complex and therefore, an increase in size due to deficient condensation of the nucleotides leading to unstable systems, has been reported for complexes comprising pDNA [72,94]. By contrast, the formation of positively charged complexes, may in principle provide many advantages: Under a certain range of N/P charge ratio is observed a contraction in the size of the complexes and efficient condensation of the polynucleotides, the positive net surface charge will favor the electrostatic interaction with the cell membrane and the presence of a high number of amino groups will potentiate the mechanism of “proton sponge” for endolysosomal release and efficient unpacking of the genetic material [72].

The design of successful gene delivery systems entails to find the fine balance of the parameters described above. Indeed, complexes with optimal association and dissociation capacity that attain the highest rates of transfection, can be achieved under specific values of chitosan’s *M*_w_ and DA, and at the optimal N/P charge ratio. The DA affects the charge density of chitosan and thus the binding sites to nucleotides, while the length of the chitosan influences the condensation efficiency of the cargo and together with the N/P ratio are important in balancing condensation, protection and intracellular release, to ensure efficient nucleic acid delivery and high rate of transfection [48,69,90,100]. For instance, it has been found that a high molecular weight chitosan yielded a higher transfection efficiency of DNA at a low N/P ratio, whereas a low *M*_w_ chitosan was required at higher N/P ratio to completely form the complexes [75,90]. On the other hand, chitosan of high DAs requires elevated values of N/P charge ratio to fully condense nucleotides (DNA and siRNA) [72,90,91].

Table 1 summarizes the reviewed studies on the role of different chitosans to complex with DNA or RNA and the major findings of each.

In vivo applications using chitosan based non-viral gene delivery are limited due to lower transfection efficiencies in comparison with viral systems. The conflicting evidence from the various studies regarding the role of the *M*_w_, DA and N/P of chitosan on the transfection efficiency by different type of nucleic acids reveals the overall dearth of fundamental understanding on the mechanisms of interaction at molecular level between chitosan and nucleic acids. Computational simulations using molecular dynamics may turn out to be extremely insightful in shedding light in this regard. This knowledge is key to a more rationally-based design of effective gene delivery systems.

## 5. Characterization Techniques of the Biophysical Properties and Biological Performance of Chitosan–Polynucleotide Complexes 

Chitosan–polynucleotide supramolecular complexes are characterized in their biophysical properties, namely size distribution, zeta potential, morphology, binding affinity, buffering capacity, colloidal stability, cytotoxicity, and transfection efficiency. A wide range of characterization techniques are used for this purpose. In this section, we address the main techniques with examples of their use in the study of chitosan–polynucleotide systems.

### 5.1. Agarose Gel Electrophoresis

Electrophoresis is a routinely used technique to separate macromolecules based on their size and charge—especially proteins and nucleic acids. Charged molecules can migrate in presence of an electric field towards the polarity of the system used. Nucleic acids have a consistent negative charge from the phosphate groups present and they migrate towards the anode (positive pole). The migration speed is determined by the molecular weight of the nucleotides. Visualization is carried out using ethidium bromide or SYBR Green under 300 nm UV light. Agarose gel electrophoresis can be used to study binding affinity of chitosan to nucleic acids during complexation [84], release capacity, protection against endonucleases [73] and stability [84,109].

### 5.2. Dynamic Light Scattering (DLS)

The technique is based on probing the time fluctuations of macromolecules and colloidal particles subjected to Brownian motion when dispersed in a solvent. Then, a monochromatic light beam (HeNe) hits the solution, causing a Doppler Shift of the light. DLS measurements allow evaluating the ability of a macromolecule to diffuse in solution. This is determined by the mutual translational diffusion coefficient (or diffusivity). The diffusion coefficient, according to Fick’s first law of diffusion, relates the concentration gradient of a solute in a solvent along an axis. Using an autocorrelation function, together with the diffusion coefficient, it is possible to calculate the mean hydrodynamic radius of the particle assuming spherical geometry and the polydispersity of the particle size distribution. The Stokes-Einstein equation (Equation (1)) relates the diffusion of spherical particles through a liquid and allows the determination of the hydrodynamic radius (*R*_H_) of the scattering particles in a medium of known viscosity (η) at specific temperature (*T*) assuming Brownian motion (*k*_B_) [110]:

(1) $$D_{S}\;=\;\frac{k_{B}T}{6\pi \eta R_{H}}$$

In Equation (1), *D*_S_ defines the self-diffusion coefficient measured by DLS, and this leads to correlate that small particles will move faster than larger ones, and therefore will have high diffusion coefficients [110,111,112]. This technique is useful for particles with size lower than a micron. Generally, the average size diameter of chitosan-based complexes is determined by DLS. Complexes formed using a series of different chitosans range from few nanometers to less than a micron [81,93,101,113].

### 5.3. Zeta Potential 

The zeta potential can be determined from the electrophoretic mobility and determination of the velocity of the particles using Laser Doppler Velocimetry (LDV). The electrophoretic mobility is the velocity of a charged particle relative to the liquid it is suspended in under the influence of an applied electric field. When the voltage is applied to the electrodes, the charged particles will migrate towards the oppositely charged electrode with a determined mobility. Using the Henry equation (Equation (2)) the electrophoretic mobility is converted to Zeta potential to enable comparison of materials under different experimental conditions.

(2) $$U_{E}\;=\;\frac{2\varepsilon \xi f_{\left(K_{a}\right)}}{3\eta }$$

where $U_{E}$ is the electrophoretic mobility (m^2^·V^−1^·s^−^^1^), ε is the dielectric constant of the medium (F/m), ξ is the zeta potential (mV), $f_{\left(K_{a}\right)}$ is Henrys function and η (poise) is the viscosity of the medium.

The zeta potential gives an indication of the potential stability of the particles. If the particles possess a large value of zeta potential, then they will tend to repel each other and there is no tendency to flocculate. However, if the particles have low zeta potential values the repulsive forces are very small in magnitude and the system turns unstable and inexorably precipitates. This technique has also been utilized to characterize the stoichiometry of complex formation between chitosan and DNA [95,101,114].

### 5.4. Transmission Electron Microscopy (TEM)

**Figure 5:**
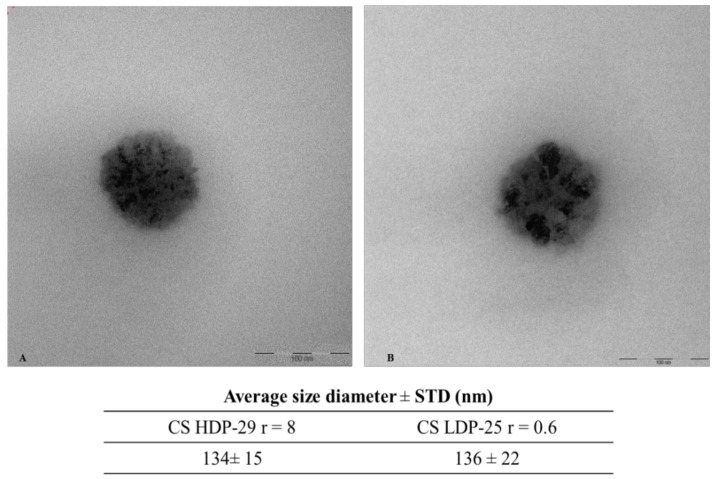
Representative TEM images of complexes containing: (**A**) CS HDP-29 N/P = 8; and (**B**) CS LDP-25 N/P = 0.6 stained with uranyl acetate. The embedded table shows the measured diameter of the complexes using ImageJ v1.49n (*n* = 8; mean average ± SD). Source Santos-Carballal et al. Scientific Reports 5, Article number: 13567 (2015) doi:10.1038/srep13567 [81], licensed under a Creative Commons Attribution 4.0 International License.

TEM operates on the same basic principles as the light microscope but with the contrast is based on the use of electrons. The electrons are emitted from a tungsten filament and travel through a 2 m column under vacuum conditions to avoid their scattering. During their trajectory, the electrons are accelerated at a high voltage (100–1000 kV) to a velocity approaching the speed of light (0.6–0.9 c); the associated electron wavelength is five orders of magnitude smaller than the light wavelength. The resolution obtained is then many orders of magnitude better than with normal microscopes, since the resolving power is directly proportional to the wavelength of irradiation; the faster the electrons are accelerated, the shorter their wavelength and higher the resolution. These characteristics enable materials imaging with the finest details of internal structure and determination at the atomic level [115,116]. TEM have been used to analyze the morphology, size and density of chitosan–DNA complexes. In the literature is described that the complexes can present different structures such as spherical shape [117], toroids and globular particles [47,118]. In Figure 5, a micrograph for chitosan–has-miRNA-145 polyplexes obtained with two distinct chitosans and at varying N/P ratios is shown. The micrographs reveal that the simple complexation of the two biopolymeric structures from aqueous solution, leads to the formation of spherical micro-heterogeneous structures, reminiscent of the morphology and size range of adenoviruses.

### 5.5. Atomic Force Microscopy (AFM) 

AFM operates under the principle of a spring-like device called cantilever, with a spring constant weaker than the equivalent spring between atoms (<~10 N/m). This is used to sense Ångström-size displacements while a tip attached to the cantilever is lowered to the sample and moves along its surface, thus creating a deflection. Given the small force applied, it would not be enough to push the atoms out of their atomic sites. The applied force can be magnetic, electrostatic, or the result of interatomic interactions between the tip and the sample. Regardless of this, all AFM instruments have five essential components [119]: (i) a sharp tip, usually 10–20 nm made commonly of silicon nitride (but can also be of ~5 nm made of carbon nanotubes), mounted on a soft cantilever spring; (ii) a way of sensing the cantilever’s deflection; (iii) a feedback system to monitor and control the deflection (and hence, the interaction force); (iv) a mechanical scanning system (usually piezoelectric) that moves the sample with respect to the tip in a raster pattern; and (v) a display system that converts the measured data into an image. Two different AFM techniques are known to generate the image. In the “contact mode”, the distance between the sample and the tip is adjusted to keep the cantilever at constant deflection. The voltage supplied to the piezoelectric tube to maintain the constant deflection is used to generate the image. This technique is not adequate for imaging soft materials. A more widely used technique, known as “tapping mode” or “non-contact mode” is used to image fragile and soft materials including biological samples. In this case, the cantilever oscillates in a sinusoidal manner at its resonance frequency. The amplitude of the oscillation is dampened when the tip approaches the sample, and the data are relayed to the piezoelectric tube, to ensure the amplitude of the vibration is kept constant. The advantage of the tapping mode is that lateral forces are eliminated and it allows to image weakly absorbed samples. 

**Figure 6:**
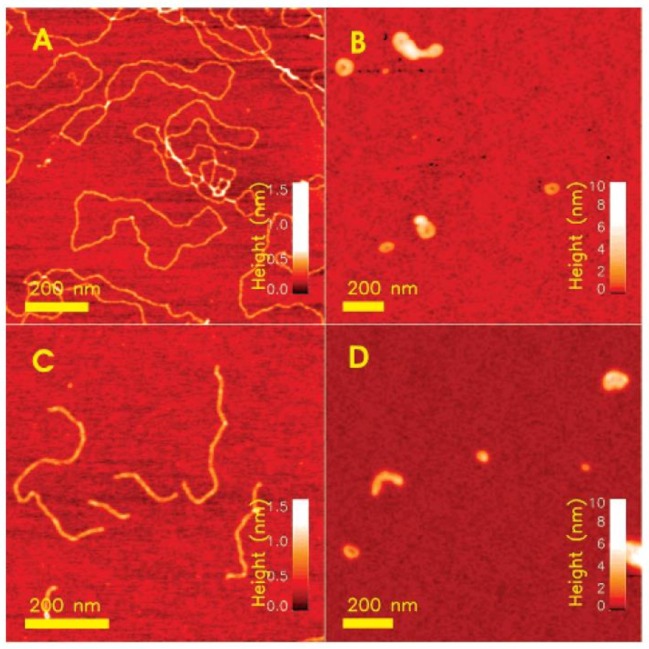
Tapping mode AFM height topographs of: uncomplexed pBR322 (**A**); and linear DNA (**C**); alongside with complexes of these formed when mixed with the chitosan C (0.01,162) (**B**, **D**) cDNA 4 µg/mL and N/P = 1. Reprinted with permission from Danielsen et al. (2004) Biomacromolecules 5, 928–936 [120]. Copyright 2004 American Chemical Society.

AFM is a high-resolution microscopy technique that enables to image atomic-scale topologies. It has been instrumental to elucidate the morphology of diverse structures of chitosan–DNA and chitosan–siRNA complexes formed under different conditions. Tapping-mode AFM has been utilized to demonstrate that the complexation of both linear and circular pDNA (pBR322) with chitosan (*M*_w_ 162 kDa, DA 10%) yields a blend of toroidal and rodlike structures [120]. Differences in the toroid-to-rod ratios were associated to the conformation of the DNA for complexes at N/P = 1.0 (Figure 6). A larger fraction of globular structures appeared for the linear DNA than for the pDNA. Quantitative analysis of the formed structures revealed that the fraction of toroids/rods decreases with decreasing charge density of chitosan (i.e., increasing DA). High and low *M*_w_ chitosans yielded the same type of structures.

It has been shown by tapping-mode that chitosan (*M_w_* 3 and 150 kDa DA 43%)–linear DNA complexes, results in the formation of “tadpole” and “question mark” structures [114]. Another study gave evidence of the formation of irregular shaped nanoparticles for chitosan (*M*_w_ 50 kDa DA 4%) linear DNA, thus confirming the capacity of chitosan to compact DNA [101]. Other studies have documented the formation of ionically gelled nanoparticles of chitosan (low *M*_w_ and DA ~15–25%) with sodium deoxycholate to associate pDNA (pCMV-GLuc, 5.7 kbp, load 5%). Spherical morphology for these systems was imaged by tapping mode AFM [121]. In addition, the same technique has been utilized to visualize the topology of complexes formed by a series of chitosans (*M*_w_ 8.9–173 kDa, DA 5–46%) with siRNA (21 bp) N/P = 50 deposited in a mica surface [4]. The formation of different type of structures spanning rod- or circle-shaped nanoparticles and open structures was evidenced for the different chitosans.

### 5.6. Surface Plasmon Resonance (SPR)

**Figure 7:**
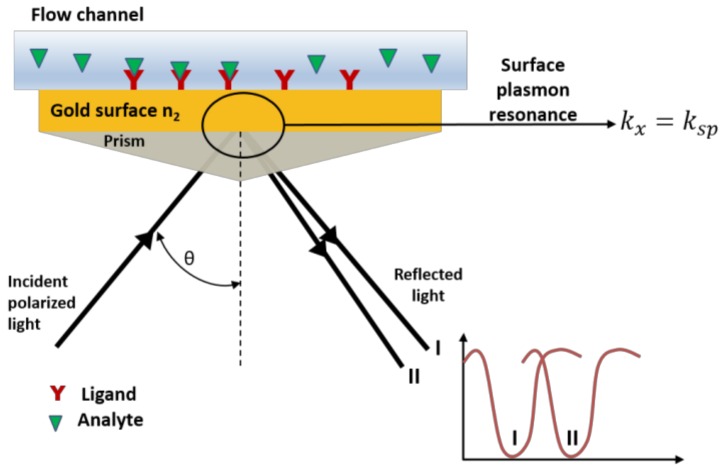
General principle of SPR, where n2 is the refractive index of medium with lower refractive index, E is the evanescent field amplitude, *k*_sp_ is the wavevector of surface plasmons, and *k*_x_ is the wavevector of photon.

SPR is a technique used to characterize the affinity events between an analyte and a ligand, allowing real-time monitoring of binding kinetics. Briefly, a beam of polarized light propagates in a medium of high refractive index (e.g., a prism) with total reflection until it hits a gold-coated surface with low refractive index (n2). The electromagnetic field component penetrates the surface for few micrometers and the intensity of the resulted polarized light is attenuated. Electrons oscillate within the surface of the conductor (gold surface), and the quantization of this oscillation is called plasmon. Once the surface is irradiated with the polarized light, the surface plasmons can couple with the protons of the polarized light, and this phenomenon is called surface plasmon resonance (Figure 7). This occurs when the condition that the wavevector of the photon (*k*_x_) is equal to the wavevector of the surface plasmon (*k*_sp_) is satisfied. The *k*_sp_ is determined by the refractive index of the conductor and the *k*_x_ depends on the wavelength of the polarized light and its angle of incidence. SPR probes the variation in the refractive index of this transducing layer induced by the adsorption or chemical reaction of an analyte. The change in the refractive index is followed by measuring the intensity of the reflected light at different angles of incidence (Figure 7) [122,123,124].

The final response units (RU), related to the change in the refractive index, are directly associated to the concentration of analyte absorbed on the surface. These values can be applied to the Langmuir Adsorption Isotherm model, which describes 1:1 interaction where one ligand molecule interacts with one analyte molecule, and the chemical reaction for the monolayer adsorption can be represented as follows [125]:

(3) A+B↔AB

where *AB* represents a solute molecule bound to a surface site on *B* during the reaction with the analyte *A*. The dissociation constant *K_D_* for this reaction is given by:

(4) $$K_{D}\;=\;\frac{k_{d}}{k_{a}}\;=\;\frac{\left[A\right]\left[B\right]}{\left[AB\right]}$$

During the reaction, the fraction of *B* that has reacted at certain time can be described as follows:

(5) $$Fraction\;B\;=\;\frac{\left[A\right]}{K_{D}+\left[A\right]}$$

When the reaction has taken place to the half of its extension and the fraction of *B* left is only ½, the condition of $K_{D}\;=\;\left[A\right]$ is satisfied and the dissociation constant can be related to the amount of analyte injected [125]. The sensitivity of SPR method to probe interactions between a given ligand-analyte system can reach the order of fM [126].

**Figure 8:**
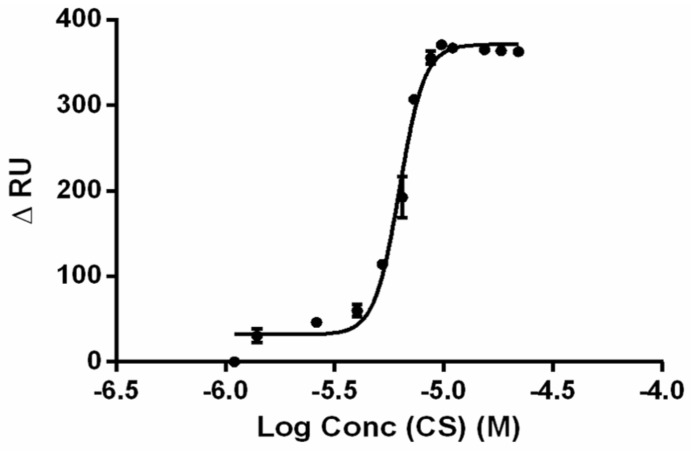
Saturation curve for hsa-miR-145-5p with HDP-12. Acetate buffer (35 mM, pH 5.1/10 mM NaCl) (*n* = 2). Source Santos-Carballal et al. Scientific Reports 5, Article number: 13567 (2015) doi:10.1038/srep13567 [81], licensed under a Creative Commons Attribution 4.0 International License.

SPR has been used to study the interaction between polymer-DNA complexes based on PEI or dendrimers and hyaluronic acid as a model glycosaminoglycan [127]. González-Fernández et al. used SPR to evaluate the binding affinity of RNA anti-tobramycin aptamer with different modifications to understand its influence on affinity towards its target molecule, tobramycin [128]. More recently, SPR has been used to study the dissociation of complexes between microRNA and a family of chitosan, e.g., two molecular weights and various degrees of acetylation to comprehend the influence of these factors in the binding [81]. Figure 8 illustrates a representative SPR curve obtained between chitosan of DA 12% *M*_w_ 25.5 kDa. The KD values obtained for the different chitosans in this series ranged from 5.93 to 31.76 µM, revealed that the chitosan found with greater transfection efficiency (*M*_w_ ~29.2 DA 29%), had a KD ~11.84 µM, thus confirming that the affinity of binding must be neither too high nor too low. 

### 5.7. Isothermal Titration Calorimetry (ITC)

**Figure 9:**
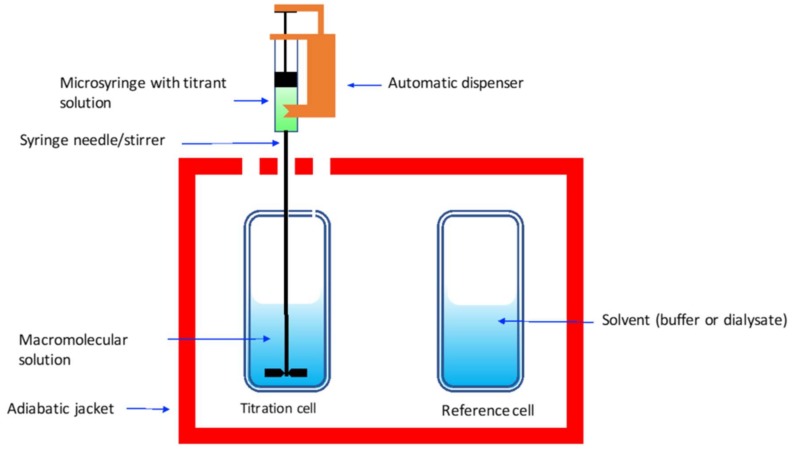
Schematic view of an isothermal titration calorimeter.

ITC is a technique that enables to characterize the complete thermodynamic profile of molecular interactions and molecular complex formation. This profile includes conformational changes, hydrogen bonding, hydrophobic interactions and electrostatic interactions, and how these interactions are interpreted at the molecular scale. An ITC instrument is composed of two identical cells made of a good conducting material and surrounded by an adiabatic jacket (Figure 9).

Very sensitive thermocouples are used to measure the differences in temperature between the reference and the titration cell. These are filled, respectively, with the solvent (e.g., 0.05% acetate buffer pH 5.0) and the measured solution (e.g., the chitosan solution). Solution with well-known concentration of the titrant (e.g., DNA solution) are placed on the automatic micro-syringe and small aliquots are injected into the titration cell. The titrant syringe functions as stirrer (e.g., 200 to 300 rpm). 

For a bimolecular interaction between chitosan and a polynucleotide, the equilibrium binding constant, *K*a, between a free chitosan molecule and a free binding site of DNA or RNA, is represented as the ratio of the molar concentration of the complex [*AB*] to the product of the molar concentrations of free binding sites in DNA and free chitosan, [*A*] and [*B*], respectively.

For the reaction [A]+[B]→[AB]

(6) $$K_{a}\;=\;\frac{\left[bound\;chitosan\right]}{\left[free\;binding\;sites\;DNA\right]\left[free\;chitosan\right]}\;=\;\frac{\left[AB\right]}{\left[A\right]\left[B\right]}$$

This model assumes independent binding sites. The complete characterization of a given macromolecular complex by ITC requires separating the enthalpic and entropic contributions of the binding process. Hence, ITC measures *K*a, enthalpy change Δ*H*, binding stoichiometry and the entropy of change Δ*S*. ITC has been used to characterize the interactions of chitosan (*M*_w_ 7.4 to 153 kDa, DA 2–28%) and 6.4 kb plasmid EGFPLuc [129], as well as between chitosans of fungal origin (*M*_w_ 44 to 143 kDa and DA 14% to 22%) and siRNA luc GI3 [98]. A conclusion from these ITC studies regarding the influence of chitosan *M*_w_ and DA on binding affinity to DNA was that chitosans of low *M*_w_ bind more strongly to DNA at high charge density (i.e., low DA), whereas chitosans of high *M*_w_ bind more strongly at lower charge densities (i.e., high DA) In previous studies using ITC, it has also been possible to address the influence of the inclusion of hyaluronic acid on chitosan–siRNA complexes [102]. Both chitosan–siRNA systems (in the absence and presence of hyaluronic acid) show that the formation of the complexes is an endothermic process. This is ascribed to the delocalization of water molecules around the charge compensated regions and thus, the shuffling of condensed counterions. 

### 5.8. Dye Displacement Titrations

Dye displacement titration is based on the quenching of fluorescence using fluorescent dyes as reporter molecules. Dyes need to exhibit relative enhancement of fluorescence upon binding to nucleotides as compared with that obtained when the dye is in solution [92,130]. Cyanines such as ethidium bromide, SYBR Green^®^ and SYBR Gold^®^ present high values of fluorescent quantum yield when they are intercalated into the bases of nucleic acids forming complexes [131,132]. The displacement of the dye from their complexes with nucleotides upon analyte titration will result in a decrease of fluorescence. The extent of fluorescence decrease is directly related to the binding between the ligand and the analyte. This titration assay is generally useful for establishing DNA/RNA binding affinity to different molecules, sequence selectivity, and binding stoichiometry [92,130]. The assay is not exclusive to titrations with small molecules; it has been used with a variety of ligands, including proteins and polymers [66,131,132,133]. Recently, a protocol based on the fluorescence determination of free pDNA with SYBR Gold^®^ to quantify the percentage of pDNA complexation with trimethyl chitosan has been documented [134].

### 5.9. Circular Dichroism (CD)

Circular dichroism spectroscopy is an optical technique that evaluates the interaction between chiral molecules with circularly polarized light. Optically active molecules (e.g., asymmetric molecules) containing chromophore groups generate a deviation in circularly polarized light, resulting in a variation in the absorption of the left and right-hand components of the light. The conformational changes in the structure of macromolecules can be evaluated using CD. This spectroscopy is very sensitive to the secondary structure of polypeptides, proteins and nucleic acids [135,136,137]. CD of nucleic acids is generated by the asymmetry of the backbone that is composed by chiral sugars. The additional π→π* interactions that lead to the helical arrangement of its bases generate electronic transitions within 200–320 nm, being the stacking geometry of the bases the main component of the CD spectra of nucleic acids [136,137]. Therefore, DNA and RNA may experience conformational changes as a result of the binding process to different compounds that can be tracked using CD spectroscopy [138].

### 5.10. Confocal Laser Scanning Microscopy (CLSM)

CLSM is a classical technique for a wide range of investigations in the biological and medical sciences. CLSM uses a focused laser beam to scan the three-dimensional volume of cells or tissues labeled with specific fluorophores. The images are obtained at a higher resolution with depth selectivity compared to conventional optical microscopy or fluorescence microscopy [139]. It has been extensively used for studying the intracellular trafficking of a broad range of nanoparticles during their interaction with cells [38,58,140] and in addition for the exhaustive comprehension of chitosan as gene delivery system [72,81]. The advantages of using CLSM include that, as the sample is exposed to the laser beam, it can be imaged many times; the ability to control depth of field; and elimination or reduction of background information. However, the main disadvantage lays on the limited number of excitation wavelengths available with common lasers [139].

### 5.11. Digital Holographic Microscopy (DHM)

DHM is a label-free imaging technique that allows visualization of transparent cells. The use of conventional techniques such as light bright field microscopy to observe cells can only generate small changes in the amplitude of light. In turn, DHM allows to multi-focus quantitative phase imaging of living cells. Furthermore, the cell volume and mass can be determined after determination of the refractive index of the cells. This provides real-time information about a possible cell swelling or shrinking in living cells. Based on these features, DHM has been successfully applied in several biomedical applications [141].

A basic setup for DHM is composed by an illumination source, most likely a coherent laser (monochromatic) to produce interference. This is followed by an interferometer and a digitizing camera. The monochromatic light used is divided in two beams to go through the reference and the sample. After illumination, the waves front from the sample and from the reference are superposed by a beam splitter to generate the interference pattern, e.g., the hologram. A single hologram is used to reconstruct the optical field and with that to generate the image using an appropriate algorithm to reconstruct the digital image [141,142].

DHM is an interferometric and non-invasive method able to distinguish cellular morphological changes. It is related to biophysical parameters and it can reveal absolute values of cell volume, dry mass, tissue density, transmembrane water transport and cell death [143,144]. DHM presents many advantages such as continuous cell monitoring over time and enabling to get insights about cellular processes such as migration, proliferation, death and differentiation. Ongoing studies in our laboratories are addressing the use of DHM in gene delivery by observing the motility of the cells in a migration study after treatment with chitosan particles containing miRNA. 

### 5.12. Real-Time Quantitative Reverse Transcription Polymerase Chain Reaction (RT-qPCR)

Quantitative RT-PCR is a sensitive and powerful tool for analyzing RNA and is the method of choice for detection and quantification. The initial step in RT-PCR is the synthesis of the complementary DNA (cDNA) of the isolated RNA. The reaction is carried out using a reverse transcriptase (retroviral enzyme) and an oligonucleotide primer. The oligonucleotide primer anneals to the RNA and, therefore, will initiate the synthesis of cDNA toward the 5’ end of the mRNA through the RNA-dependent DNA polymerase activity of the reverse transcriptase. Primers can target a specific gene or be non-specific (random hexamer primers) that present all possible nucleotides combinations and bind to all RNAs sequences. The synthesis of cDNA is the source of variability in the results, since the reverse transcriptase enzyme is sensitive to salts or alcohols remaining from the RNA isolation [145]. Afterwards, the cDNA is amplified by PCR. PCR is generally constituted by steps of denaturation, annealing and elongation that are programmed in cycles. The number of cycles depends on the amount of target present and the efficiency of the reaction. The quantification and detection of the target gene can be carried out using two main techniques: “end-point” that measures the final concentration at the end of the reaction and the “real-time” that monitors the formation of the product during each cycle of the polymerase chain reaction. The quantification technique for “end-point” determinations can be done using fluorescent intercalating dyes. The “real-time” quantification uses primers and probes separately. This can be carried out using Taqman-DNA^®^ polymerase, by using its 5’ exonuclease activity to cleave and displace the probe which leads to sequence-specific fluorogenic hydrolysis, fluorescence is no longer quenched and can be quantified [145,146]. The number of cycles required to reach a threshold of fluorescence is expressed as the Ct value. The “real-time” quantification requires the normalization to a reference gene or housekeeping gene for internally controlling the variation of the target gene. The analysis of the data is done by the relative expression of the housekeeping gene to the 2-ΔΔCt [147].

## 6. Plasmid DNA Delivery

At the molecular scale, pDNA can be considered a prodrug, given that, once it reaches the nucleus, it drives the synthesis of a therapeutic protein. Moreover, plasmids can also be considered as vaccines.

The Food and Drug Administration (FDA) defines DNA vaccines as “purified plasmid preparations containing one or more DNA sequences capable of inducing and/or promoting an immune response against a pathogen”. A DNA vaccine consists of DNA plasmid (pDNA) containing a transgene that encodes the sequence of a target protein from a pathogen under the control of a eukaryotic promoter. Although the idea of using DNA plasmids for vaccination dates from more than 20 years ago, only four DNA veterinary vaccines are approved, and no DNA vaccines for humans are available yet [148]. One reason for this low success rate might be the weak immune response that DNA vaccines alone showed in different clinical trials. Nevertheless, genetic vaccines have many advantages over conventional ones. DNA vaccines can also induce both long-lasting cellular and immune responses but do not revert into virulence, hence they rise fewer safety concerns. Another advantage is that DNA vaccines do not integrate into the genome. pDNA is a double stranded DNA molecule, containing up to 200 kbp, and they can exist in three distinct topological configurations: (i) compact supercoiled from; (ii) relaxed open circular form; and (iii) linearized form. The FDA requires that more than 80% of the pDNA be in the supercoiled form for application in pDNA vaccines for infectious diseases applications [149]. It has been shown that the supercoiled form of DNA has greater transfection efficiency than its open circular and linear counterparts [150,151].

pDNA cell transfection entails cellular uptake, endolysosomal escape, pDNA unpackaging, intracellular trafficking and nuclear import. The potential of chitosan and its derivatives for dDNA delivery has been widely documented. Among the pioneering studies on the use of chitosan polyplexes as an effective non-viral pDNA immunization vector gave proof of concept of the oral delivery of the plasmid pCMVArah2 encoding for the dominant peanut allergen to hypersensitive AKR/J mice. Four weeks after administration, the animals that were treated with chitosan/pDNA nanoparticles exhibited greater levels of IgA, consistent with the induction of a mucosal immunization response. In addition, the immunized mice, had less severe and delayed anaphylactic responses after intraperitoneal challenge with peanut protein Arah2 [152]. This study was the first one to show the potential of chitosan as a non-delivery vector for pDNA mucosal immunization. A pitfall in this work though, was that the chitosan used was only partially characterized as having a very high *M*_w_ (390 kDa), while the DA was not given neither in the paper nor in the preceding one.

Chitosan–pDNA polyplexes, chitosan-based nanoparticles crosslinked by ionic gelation, or either of these mixed with other biopolymers (e.g., hyaluronic acid) or beta cyclodextrins, have been reported in both in vitro and in vivo studies focused on pDNA delivery. Chitosan is a particularly attractive biopolymer for mucosal administration of pDNA vaccines. Recent studies have shown that chitosan (low *M*_w_ (undefined), DA 2%) ionically crosslinked with sodium tripolyphosphate, can be used to deliver the supercoiled topoisoform of pcDNA-FLAG-p53 plasmid [151]. To the best of our knowledge, this is the only study that has addressed the influence of chitosan on the conformation of DNA. This is an aspect that deserves much more attention in establishing the role of chitosan’s structure on the conformational preference of both pDNA and siRNA.

## 7. RNA Interference Machinery

In cells exists a process known as RNA interference (RNAi) that regulates the degradation of RNA in a highly sequence-specific manner [153]. In general, RNAi process is activated by introduction of double stranded RNAs (dsRNAs) into cells, promoting the degradation of mRNAs, controlling mRNA translation, and therefore interrupting protein synthesis [153]. RNAi mechanism can be triggered by either small interfering RNAs (siRNAs), which can decrease gene expression through mRNA transcript cleavage, or endogenous microRNAs (miRNAs), which primarily inhibit protein translation [154]. siRNA machinery is found in plants and bacteria, and exogenous introduction in mammalian cells can target a specific mRNA with 100% complementarity. Endogenous mRNA regulation in mammalian cells is mediated by miRNAs [154,155].

**Figure 10:**
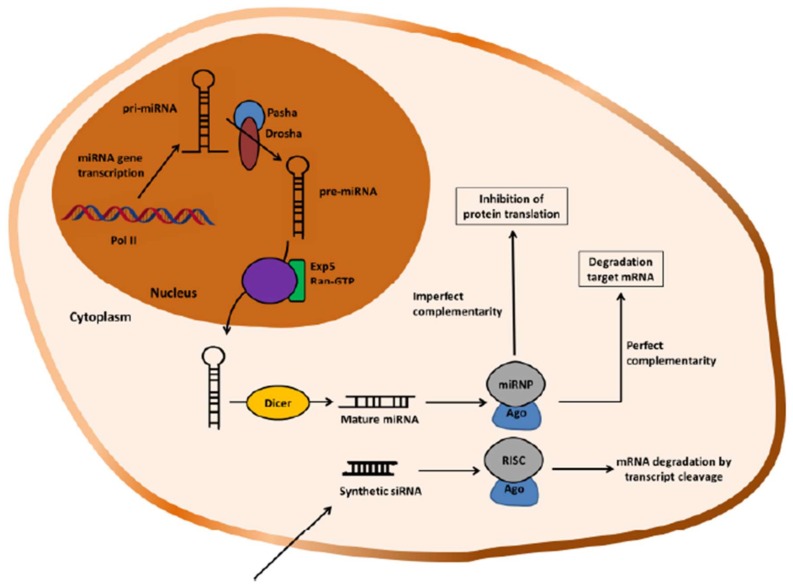
Simplified scheme of RNA interference mechanism in mammalian cells. Only processes mentioned in the text are illustrated.

In Figure 10, is shown a schematic description of miRNA biogenesis and the RNAi mechanism mediated in mammalian cells. Briefly, the gene-silencing mechanism for miRNAs is induced by endogenous dsRNA transcribed by RNA polymerase II (Pol II) in the nucleus, producing so-called pri-miRNAs. The pri-miRNAs are cleaved to yield ~70-nucleotide pre-miRNAs by the RNase III enzyme, Drosha, and its cofactor, the double-stranded RNA-binding protein, Pasha. The pre-miRNAs are imperfect stem-loop structures, which are exported into the cytoplasm via Exportin 5 (Exp 5) and its catalytic partner Ran-GTP. These pre-miRNAs are degraded by the RNase III endoribonuclease Dicer into miRNAs constituted by 21–23 nucleotides. Afterwards, they incorporate into a miRNA ribonucleoprotein complexes (miRNPs). In the case of siRNA, their exogenous introduction into mammalian cells also leads to assemble into a ribonucleoprotein complex known as the RNA-induced silencing complex (RISC). These complexes are believed to be similar, if not identical. Argonaute unwinds miRNA/siRNA within their respective functional complexes and leads to the retention of the guided strand in the complex and at the same time the complementary strand is removed and degraded. Further, exogenous siRNA mediate sequence-specific silencing by inducing mRNA cleavage and subsequent mRNA degradation of target transcripts. In the case of miRNA, it can bind either with imperfect complementarity to its target mRNA matching with the targets 3’ untranslated region (UTR) resulting in inhibition of protein translation and/or with perfect complementarity which results in degradation of the target mRNA [25,154,156,157,158].

### 7.1. MicroRNAs Implication in Cancer: Oncogenes and Tumor Suppressors

MicroRNAs (miRNAs) are small non-coding RNAs of 20 to 24 nucleotides found in all eukaryotic cells, playing important roles in almost all biological pathways in mammals and other multicellular organisms [158]. The first reports identifying small non-coding RNA date from 1993 and 2000, where lin-4 and let-7 genes were identified in *Caenorhabditis elegans* acting as post-transcriptional repressors of their target genes when bound to their specific sites in the 3’ untranslated region of the target mRNA [159,160]. In the last fifteen years, many reports have emerged focusing on miRNA and it is estimated that they regulate ~60% of human genes, e.g., 250–500 different mRNAs, making them very powerful gene regulators [161]. MicroRNAs influence numerous cancer-relevant processes such as proliferation, cell cycle control, apoptosis, differentiation, migration and metabolism [162,163,164]. This leads to huge opportunities, for researchers looking for therapeutic targets or mimics. MicroRNAs can promote or repress cell proliferation, differentiation and apoptosis during normal cell development [165,166]. Dysregulation of miRNA expression can affect a multiple number of cell signaling pathways and in that way influence cancer onset and progression. Some miRNAs may function as oncogenes or tumor suppressors. Understanding the role of miRNAs in human malignant tumors is key in the development of new therapeutic approaches. 

Lu et al. demonstrated that miRNAs have different profiles in cancers compared with normal tissues, and those vary among different cancers [167]. The function of miRNAs in cancer pathogenesis can be studied by upregulation or downregulation of its expression. Upregulation of miRNA expression in tumors is regarded as oncogenes. Increased expression of miRNAs, would negatively inhibit miRNA-target tumor suppressor genes that control cell differentiation or apoptosis, contributing to tumor formation by stimulating proliferation, angiogenesis, and/or invasion. The causes of this dysregulation are not completely elucidated, but might be due to amplification of the miRNA gene, which would increase the efficiency in miRNA processing or increased stability of the miRNA [165,168,169,170]. The expression of some miRNAs can be as well downregulated, these miRNAs are also referred as tumor suppressor miRNAs. Tumors often present reduced levels of mature miRNAs due to genetic loss, epigenetic silencing, defects in their biogenesis pathway or widespread transcriptional repression [167]. In normal cellular process these tumor suppressor miRNAs prevent tumor development by negatively inhibiting oncogenes. Therefore, a downregulation of those consequently increases proliferation, invasiveness or angiogenesis, decreases levels of apoptosis, or undifferentiated or de-differentiated tissue, leading to tumor formation [165,168,169,170].

## 8. Rare Diseases

A rare or “Orphan” disease, as defined by the World Health Organization (WHO), is any disease or condition that affects 0.65–1% of the total global population. These diseases affect a significant proportion of the population; indeed, there have been more than 6000 types described worldwide [171]. The complex etiology of such diseases and the widely heterogeneous symptoms result in significant challenges faced by the scientific community as well as a lack of viable therapeutic options for patients suffering from these diseases. The treatment of individuals affected by rare diseases is hampered by poorly understood mechanisms slowing the development of suitable therapeutics. Advances in rare disease diagnostics, based on data from clinical trials, are the focus of gene therapy studies, enzyme replacement therapy and new drug discoveries, whose identification can be accelerated by drug repositioning. These advances have allowed the better characterization of rare diseases, especially those that are monogenic. Nonetheless, several factors have hindered therapeutic development for rare diseases such as the small market share that they represent the high cost of production new therapeutics and the potential low return on investment [172].

Addressing several rare diseases that share a common molecular etiology within a given project is especially attractive as the majority of rare diseases have an underlying genetic cause [173]. Undertaking drug repurposing screens for a variety of rare diseases that share a common molecular etiology will expedite drug discovery for these conditions. For the rare disease population, gene therapy is a hopeful inspiration. 

### 8.1. Cystic Fibrosis

Cystic fibrosis (CF) is a genetic disease caused by mutations in the cystic fibrosis transmembrane conductance regulator (CFTR) gene [174,175]. This encodes a cyclic AMP (cAMP) dependent channel expressed in the epithelia of many exocrine tissues including the airways, lung, pancreas, liver, intestine, vas deferens and sweat gland/duct [176]. CF is the most common lethal autosomal recessive disease in people from Northern European descent affecting approximately 70,000 individuals worldwide (www.cff.org). Although medical advances in recent decades have prolonged the average life expectancy for CF patients to ~40 years, there is still no cure for this devastating disease [177].

The CFTR gene encodes a chloride and bicarbonate channel. In addition to this, CFTR is proposed to regulate the function of other membrane proteins including the epithelial sodium channel (ENaC) [178]. CFTR and ENaC play the most important role in maintaining homeostasis by controlling the movement of water through the epithelium, thus regulating the hydration of the epithelial surface in many organs, but predominantly in the airways. Therefore, epithelial CFTR dysfunction leads to airway surface liquid volume depletion due to an imbalance between CFTR–mediated Cl^−^ secretion and ENaC-mediated Na^+^ absorption [179].

#### 8.1.1. Pathophysiology of CF

Mutations in CFTR cause abnormal ion transport in the epithelium of several tissues, which results in the production of abnormally thick and sticky mucus that blocks the organ and is responsible for CF pathology. CF encompasses a wide range of symptoms; one of the earliest markers of the disease is the meconium ileus affecting a number of CF newborns [180]. Another symptom used for early diagnosis of CF is the salty sweat caused by defective salt reabsorption in the sweat ducts [181]. Furthermore, approximately 85% of CF patients are pancreatic insufficient whereby thick, dehydrated secretions cause occlusion of the pancreatic ducts and prevent the release of the digestive enzymes into the intestines [182]. This can be managed by controlling food intake and incorporating pancreatic enzyme supplements, minerals and fat-soluble vitamins into the diet [183,184]. Older patients can suffer from the destruction of the islets of Langerhans and reduced insulin production, leading to diabetes mellitus [185]. Liver disease and infertility are other common manifestations of CF. In fact, around 98% of male CF sufferers are infertile, mainly due to congenital bilateral absence or blockage of the vas deferens [186]. However, the main cause of morbidity and mortality in CF is lung disease, with lung malfunction and pulmonary failure [187,188]. The loss of chloride secretion from CFTR deficiency results in changes in osmotic pressures and electro-neutrality which likely lead to excessive sodium and water absorption [189]. The production of sticky mucus in the lumen of the lungs impedes mucociliary clearance [190]. Clinically, this manifests as chronic inflammation and recurrent bacterial infections, commonly by pathogens such as *Pseudomonas aeruginosa* and *Staphylococcus aureus* [188]. Mucus and inflammatory cells cause bronchiectasis and the continuous cycle of infection and inflammation lead to the progressive destruction of lung tissue [191].

### 8.2. CFTR Channel 

Located on the long arm of chromosome 7 (7q31.2), the CFTR gene spans around 250 kb of DNA [192]. Consisting of 27 exons of various sizes and producing an mRNA of around 6.2 kb, the gene encodes a single polypeptide chain of 1480 amino acids with a predicted molecular weight of around 168 kDa [174,193]. The observed sequence of the CFTR protein has led to a proposed structure that shows similarity to proteins in the family of ATP-binding cassette (ABC) transporter [194]. The CFTR protein spans the apical membrane of epithelial cells and consists of distinct structural domains; two membranes spanning domains, two nucleotide binding domains and a regulatory domain. These nucleotide binding domains contain conserved motifs for ATP binding and hydrolysis. Both, the regulatory domain region, as well as the long N- and C-terminal extension, is unique in CFTR. The N- and C-terminal are about 80 and 30 residues in length, respectively. Phosphorylation of the R domain is carried out by PKA, PKC and CK2, a process which is dependent on the presence of intracellular ATP [195,196]. This event is important for regulating the opening of the CFTR channel. The dephosphorylation of the R region is thought to be brought about by phosphatases and has an inhibitory role on CFTR activity [197]. Small conformational changes may only be needed to bring about channel opening, and modest structural changes have been observed upon phosphorylation and the binding of ATP. Many other proteins including PDZ-interacting proteins and STAS domain interactors are believed to be important for the regulation of CFTR activity [198,199]. 

#### 8.2.1. CFTR Mutation

Almost 2000 distinct CFTR mutations have been described thus far by researchers working in the field of CF genetics [200]. However, by far the most common mutation described is a three-base deletion that removes a phenylalanine at position 508 (F508del) of the CFTR protein, which is the cause of disease symptoms in 85% of patients worldwide [201]. The scientific community has organized all known CFTR mutations into six classes according to their deleterious effect on the protein [176]. Classes I–III are associated with no effective CFTR function, such as total or partial lack of production, defective processing or missense mutations. Classes IV–VI show residual function, issues with pre-mRNA splicing or stability at the plasma membrane. There is now a growing consensus that CFTR mutations may also provide a scientific basis for mutation-specific corrective therapies and are accordingly grouped into seven classes depending on their functional defect [201]. Those mutations in Class VII have been defined as any which cannot be corrected through pharmacological means. A large proportion of ongoing studies now aim to apply the same therapeutic correction to the basic defect in each class.

### 8.3. Cystic Fibrosis: Implication in Gene Therapy

#### 8.3.1. Treatments for CF: Drug Therapies 

CF is a disease that may manifest with various physiological abnormalities, thus creating a diverse CF population. One main challenge with treatment of CF is therefore identifying which of these abnormalities merits more attention to achieve the best outcome [202]. The major factor linked to mortality, such as pulmonary disease, nutritional deficiencies and CF related disease could be a crucial point in the optimal management of the patients. Nutritional deficiency is linked to pulmonary function decline, and early intervention to improve nutrition is associated with better clinical outcomes, including increased survival. Furthermore, nutritional supplements and pancreatic enzyme replacement therapy have been essential in patients of all ages [203]. With improved nutritional status, CF patients have improved outcomes with respect to pulmonary symptoms. Since pulmonary disease pathogenesis in CF is a complex process associated with multiple abnormalities in the respiratory tract, laboratories around the world have characterized several strategies to restore CFTR function in the airways.

Personalized protein therapy targeting specific mutation classes allows the individualization of treatment, thus anticipating which drug will be effective in one patient versus another [204]. Administration of aminoglycoside like gentamycin has shown benefits in patients with CF [205], but pre-clinical studies have demonstrated that high-dose aminoglycosides could induce a lack of potency and side-effects [206]. Therefore, a new compound named Ataluren or PTC124, with a similar mechanism of action as aminoglycosides but lacking their antibiotic properties, has been developed [207].

Chemical compounds called “correctors”, agents that could prevent the degradation of the CFTR protein in the cell, have been developed [208]. The company Vertex developed VX-809 or Lumacaftor, a corrector that may achieve this goal. Studies with bronchial epithelial cells containing the F508del-CFTR mutation have shown increased chloride transport with VX-809 [209]. Other strategies also looked at agents that increased the chloride transport activity of CFTR, molecules called “potentiators” [210]. High-throughput screening methodologies identified a potentiator named VX-770 or Ivacaftor. VX-700 is an approved CFTR potentiator that increases the open probability of the CFTR channels [211]. In addition, the combination of Lumacaftor and Ivacaftor also called Orkambi has been associated with a higher increase in chloride transport than either agent used alone [209]. Recently, Phase III trials were conducted to evaluate the efficacy and safety of Orkambi in patients with CF homozygous for F508del-CFTR mutation. The results confirm an improvement in forced expiratory volume and a reduction in pulmonary exacerbations, thus providing a benefit for CF patients [212]. Another study by Leier and colleagues has shown that the activation of CFTR can be achieved by increasing the level of cAMP in the cell using phosphodiesterase (PDE) inhibitors such as sildenafil (Viagra) [213]. Leier and co-authors used sildenafil in F508del-CFTR/wt-CFTR expressing *Xenopus laevis* oocytes and in CF and non-CF human bronchial epithelial cell lines (CFBE41o-/16HBE14o-) to investigate the mode of action of this component. Unfortunately, the necessary high doses of the drug for CFTR recovery limit its use in the clinic [213].

Alternatively, ENaC activity could be inhibited to decrease the elevated Na^+^ absorption seen in CF airways. After aerosol delivery of the ENaC inhibitor Amiloride, to the airways of CF patients in clinical trials, correction of the Na^+^ transport defect was reported, although no long-term clinical benefit was observed [214]. Early Amiloride derivatives (e.g., Phenamil and Benzamil) had limited therapeutic use due to rapid absorption and clearance from the lungs [215]. Another strategy utilizes antisense oligonucleotides (ASOs) to inhibit ENaC activity. ASOs are short synthetic DNA molecules (15–18 bases) that are complementary to specific mRNA sequences. Some studies have focused on the ASOs strategy to down-regulate the expression of the alpha-ENaC subunit in human primary nasal epithelial cells. Sobczak et al. have shown an inhibition of Na^+^ absorption through ENaC in CF tissue by about 75% and in non-CF tissue by about 66%. Furthermore, this ASOs strategy sustained ENaC inhibition for more than 72 h [216]. Hypertonic saline treatment (HTS) has been shown to possess mucolytic properties and aids mucociliary clearance by restoring the liquid layer lining the airways [217]. However, different studies have suggested that this treatment may not be suitable as a long-term strategy to slow disease progression [218]. Nonetheless, HTS could provide a safe, low-cost addition to the daily therapy of CF patients.

Most of the potential drug therapies mentioned above target a specific class of CFTR mutation and are effective only at high doses. Subsequently, these therapies are only relevant for a small number of CF patients and would not be suitable treatments for the clinic.

#### 8.3.2. CFTR Gene Therapy

Gene therapy has emerged as an alternative to conventional treatment for diseases. The discovery of the CFTR gene in 1989 created excitement for the development of CF gene therapy [219]. The delivery of genetic material, DNA or RNA, can be utilized as a promising concept for heritable diseases, such as CF with the prospect of correcting many aspects of the complex pathology [220]. The vast majority of efforts over the last 20 years have focused on developing a curative treatment targeting the basic defect rather than treating CF disease manifestations [221]. In addition, gene therapy is mutation class independent also termed “mutation agnostic” and a single treatment strategy would be suitable for all patients.

One of the most powerful genetic therapeutic technologies is gene repair, where the specific mutated bases are targeted and corrected in the genome of an individual. The emergence of the engineered nucleases such as CRISPR-Cas-9 system, zinc finger nucleases (ZFN) and transcription activator-like effector nucleases (TALEN), are encouraging, but the low efficiencies and the time-consuming selection of the repaired cells is not feasible in vivo [222,223]. Subsequently, studies have mainly focused on gene complementation approaches, where an additional copy of the wtCFTR cDNA is delivered to cells homozygous for CFTR mutations [224].

Many viral and non-viral vectors have been tested for their usefulness in CF gene therapy [225]. Viral studies using recombinant adenovirus (rAd) were a promising gene delivery vector due to the high gene transfer efficiencies observed in animal models. However, the paucity of adenoviral receptors on the apical lung surface and the severity of the host-immune response to repeat viral delivery have demonstrated a strong argument against the effectivity of adenoviral CF gene therapy [226,227]. Subsequently, another promising viral vector evaluated for the treatment of chronic lung disease is based on recombinant adeno-associated virus (rAAV). Furthermore, numerous AAV serotypes have been discovered, with the aim of increasing transduction efficiency. However, results of rAAV treatment evaluated clinically were disappointing due to the antiviral immune response activated by repeated administration [228]. In addition to rAAv and rAAV, various cytoplasmic RNA viruses have been validated for airway gene transfer. The murine parainfluenza virus type 1 (SeV), the human respiratory syncital virus and the human parainfluenza virus have been shown to efficiently transfect airway epithelial cells via the apical membrane [229,230]. Only SeV has been used in animal models in vivo and has been able to correct the Cl^−^ transport defect in the nasal epithelium of CF knockout mice [231]. However, repeated administration was not feasible and therefore the vector has not made it to clinical trials [232,233]. Most recently recombinant lentiviruses (rLV) have gained interest as they appear to evade host immunological defenses and are able to inducegene expression in non-dividing cells [234,235]. They can be modified by the addition of novel surface proteins (pseudotyping) to specifically increase the efficiency of airway gene transfer [236]. Furthermore, lentiviral vectors can be repeatedly administered to murine airways [237], which is a major requirement for the treatment of chronic diseases such as CF.

The investigation of non-viral vectors to treat chronic CF lung disease was fueled by the necessity to develop an effective long-term, repeatedly administered treatment [238]. Typically, non-viral vectors comprise circular plasmid DNA molecules manufactured from bacteria, which are then complexed with a range of cationic lipids and polymers, known as “lipoplexes” or “polyplexes”, respectively [239]. The mechanism of non-viral gene transfer is poorly understood, but it is thought that lipoplexes and polyplexes bind to the cell membrane, are endocytosed and subsequently escape from endosomes by inducing rupture of the endosomal membrane [240]. Non-viral vectors are considered less efficient than viruses due to the lack of the specific components required for cell entry that are present in viruses [241]. Nevertheless, non-viral vectors do not contain viral proteins rendering them less inflammatory and immunogenic [242]. Further advantages associated with these vectors are their easy manipulation, the possibility to manufacture them in large quantities, their possible storage for extend periods and their unlimited packaging capacity [243].

Messenger ribonucleic acid (mRNA) is another potential alternative to conventional DNA therapies. The development of mRNA-based therapeutic approaches presents several important differences in comparison with other nucleic acid-based therapies. The delivery of mRNA allows direct translation in the cytoplasm, as mRNA is not required to enter the nucleus to be functional. Furthermore, mRNA does not integrate into the genome and therefore does not pose the risk of insertional mutagenesis. The production of mRNA is also relatively simple and inexpensive [244]. Approaches with mRNA-based gene therapy and delivery by non-viral vectors bypass some of the disadvantages associated with DNA delivery [245]. The delivery of mRNA-based therapeutics offers a greater safety profile than viral and pDNA-based vectors since they do not contain bacterial sequences. RNA is recognized by Toll-like receptor (TLRs), a family of receptors that trigger the innate and adaptive immune system to deal with infections by the recognition of pathogen-associated molecular patterns (PAMPs) [246]. Both dsRNA and ssRNA are often components of viral genomes or intermediates of replication, and are therefore recognized by TLR3 and TLR7/TLR8, respectively [246,247,248]. Future studies may show whether nucleoside modified IVT mRNA will avoid the activation of human TLRs in the clinical setting.

Recently, a proof-of-concept mRNA-based functional restoration of impaired CFTR function have been demonstrated, not only in an immortalized human bronchial CF cell line, but also in primary human nasal epithelial (HNE) cells [113,249]. Transfection of the CFBE41o- cells with CFTR-mRNA restored cAMP induced CFTR currents similar to the values seen in control cells (16HBE14o-) and an almost two-fold increase in the cAMP-stimulated CFTR current after transfection using primary cultured HNE cells. The authors demonstrated that optimized CFTR-mRNA can be reduced to a minimal dose of 0.6 μg/cm^2^ in primary HNE cells, and that this dose can be persistent for a period longer than 24 h after transfection procedures [113]. These experiments successfully established a new strategy for the delivery of CFTR-mRNA directly to airway epithelial cells.

#### 8.3.3. Different Delivery Methods for CF Lung Disease

In CF pulmonary disease the opportunity to selectively target a drug to the lungs remains a fascinating option. In fact, local drug delivery may allow maximum pharmacological targeting, and thus therapeutic efficacy. Consequently, researchers continue to apply efforts to develop new inhalation devices and advanced drug delivery [250]. CFTR-mRNA aerosol administration to airways of CF patients could be delivered as shown by Rudolph and coworkers [251]. Another recent study by Hasespunch and co-authors successfully demonstrated gene delivery with magnetized aerosol comprising iron oxide nanoparticles in the lungs of mice [252]. Therefore, administration of drugs via the inhalation route is of great interest in CF treatment. The main advantages of aerosol technologies are the limited systemic toxicity, direct drug action on target site and the suitability for home therapy [253]. Potential disadvantages include the uncertainty about drug dose at the target site as well as limited information on drug interactions in the lung [254]. Another important issue in the gene delivery method is the biocompatibility and biosafety of the nanocarrier employed in the transfection procedure [255]. Lipid based formulations like cationic lipids or cationic polymers have been proved a successful method to transfect cells and to reach adequate transfection efficiency in vitro [256].

Orientating studies demonstrated promising results using lipid-based delivery by LipofectamineTM 2000 transfection reagent in primary cultured cells. However, lipofectamine presents high cytotoxicity, compromising cell viability. Thus, despite its robust high transfection efficiency [257] it is not an appropriate carrier to assess potential clinical therapies in the treatment of CF. Therefore, alternative and stable formulations using biopolymers, such as chitosan, have been assessed for their efficacy in targeting intratracheal routes. In this work, the development and optimization of an alternative non-viral mRNA system based on a biocompatible polymer, in particular chitosan, was of great interest [258]. In general, the use of natural polymers has gained increasing interest as a safe and cost-effective delivery strategy for gene material [259]. Recent studies, including our own ones, have reported improved plasmid-based gene transfer and expression in the airways using chitosan as a nanocarrier [71,105,106]. Nydert and coworkers examined the possibility of using CFBE41o as a transfection cell model for chitosan; this was the first time chitosan polyplexes were used to transfect a CF cell line [105]. Nevertheless, the search for the optimal vector for gene therapy in the hope of finding a mutation-independent cure for CF continues and represents a major challenge in CF. Recently, we described for the first time the use of non-animal source chitosan as a gene delivery vector in the context of CF lung disease which provides an alternative to classical animal-source chitosan [108].

Rather than treating CF disease manifestations, the finding of the CFTR gene identification opened the door for targeting the basic defect to correct the mutation at a cellular level. In general, gene-based therapeutics introduce nucleic acids into cells to alter the gene expression of a pathological process [260]. Thus, the delivery of a therapeutic nucleic acid (DNA or RNA) is a promising concept for an inherited single-gene defect such as CF, with the prospect of correcting many aspects of the complex pathology [261]. In addition, one therapy might be suitable to treat subjects with a wide variety of mutations, which means that a single treatment strategy would be relevant to all patients.

Since CF lung symptoms are responsible for the majority of deaths in CF, most investigators have focused their efforts on gene therapy in airways and on targeting a reduction in lung disease [262]. Several challenges were identified in pulmonary gene delivery, including the development of efficient vectors for gene transfer concerning safety requisites, the circumvention of inflammatory responses, and the maintenance of long-term gene therapeutic expression in airways [263]. Indeed, the ciliated epithelial cells that are located in the airways are the best target for CF gene therapy [264]. The expression of the CFTR protein in the airway is mostly localized in the ciliated epithelial cells and ducts of the submucosal glands [265,266]. Furthermore, the main functions of ciliated cells include the facilitation of mucus transport and the maintenance of airway surface hydration [267,268]. Therefore, ciliated cells and their properties make them a relevant therapeutic target for CFTR gene delivery.

Although it is known that the mRNA of CFTR is in low abundance in airway epithelia [269], a minor level of gene transfer of CFTR to the airway epithelia is sufficient to correct the Cl^−^ transport in vitro and in vivo [270]. Furthermore, only 10% of normal cells were sufficient to normalize the main dysregulated parameters such as Cl^−^ or Na^+^ conductance and Interleukin 8 secretion [271].

Up to now, more than 25 clinical trials which aimed to investigate the safety and sustainability of gene transfer have been completed. Most of them were of short duration and carried out with a reduced number of patients [272]. Initial approaches, which involved the direct administration of recombinant CFTR, based on conventional DNA delivery to the airway, have not been successful for several reasons, such as immunogenicity or the limited DNA packaging capacity of the vector [273,274]. Thus, no significant benefits for patients treated with viral and non-viral vectors were found [264]. Subsequently, in vitro transcribed mRNA has been pointed out as a potential new drug class to deliver genetic information [244]. Furthermore, the development of mRNA-based therapy presents several important differences in comparison with other nucleic acid-based therapies, e.g., the direct translation in the cytoplasm, the non-integration into the genome and therefore, the avoidance of the potential oncogene expression that is caused by insertional mutagenesis [260]. In addition, exogenous mRNA can be efficiently delivered into cells independently of the differentiation stage or confluence [275].

The transfer of genetic material into the appropriate target cells is the first critical step for successful gene therapy. Among the different available methods for accomplishing gene delivery, the chemical lipofection procedure has been reported to be widely efficient [34,276,277]. In the literature, it has been said that lipofection methods present high transfection efficiencies and in vitro studies showed increased levels of transgene expression in different mammalian cell types [278]. Nonetheless, a potential reduction in cell viability and immunogenicity induction restrict the use of lipofection in vivo [279].

### 8.4. Other Rare Diseases

- Lysosomal storage diseases (LSD)

LSDs are a group of more than 40 single-gene recessive genetic diseases that result in metabolic imbalances of the lysosome. LSDs, over time, result in a spectrum of symptoms and disabilities due to tissue-specific substrate accumulations [280]. LSDs are promising targets for gene therapy because the delivery of a single gene, into a small percentage of the appropriate target cells may be sufficient to impact the clinical course of the disease. In addition to these characteristics, another important one is the possibility for the “cross-correction phenomenon” of affected tissues in patients via the extra-cellular provision of recombinant forms of the deficient lysosomal enzyme [281]. Adeno-associated virus therapies, adenovirus therapies, and hematopoietic stem cell (HSC) transplant have overcome limitations associated with earlier clinical and preclinical trials, suggesting that gene therapy may be a reality for LSDs soon. At the same time, the first EU-approved gene therapy drug, Glybera^®^, has been discontinued, and other ex vivo-based therapies although approved for clinical use, have failed to be widely adapted and are no longer economically viable. In the case of aromatic l-amino acid decarboxylase (AADC) deficiency, it is also reported that some gene therapies using specific AAV serotypes, such as AAV9, could cross the blood–brain barrier to deliver genes to the central nervous system [282].

- Metachromatic leukodystrophy (MLD)

The concept of leukodystrophy (leuko-white, dystrophy-defective nutrition) refers to a very heterogeneous group of inherited diseases in which molecular abnormalities result in a defect in myelin sheath formation or maintenance, or in some cases both [283]. Metachromatic leukodystrophy (MLD) is an autosomal recessive LSD caused by the deficiency of lysosomal arylsulfatase A (ARSA) enzyme [284]. The optimal therapy for MLD would provide persistent and high level expression of ARSA in the central nervous system (CNS). Gene therapy using adeno-associated virus (AAV) is an ideal choice for clinical development as it provides the best balance of potential for efficacy with a reduced safety risk profile. Therefore, various therapeutic approaches to MLD have been tested in experimental animal models. In addition, there are several promising approaches with potential clinical translation, including: (1) enzyme-replacement therapy; (2) bone marrow transplants; (3) gene therapy by ex vivo transplantation of genetically modified HSC; and (4) AAV-mediated gene therapy directly to the CNS [285]. Initial studies with AAV-mediated gene therapy for MLD were conducted with AAV5, and then with the discovery of the non-human primate derived serotypes, second generation studies were carried out with AAVrh.10 [286,287,288]. A Phase I/II trial in early forms of MLD using intracerebral delivery of AAVrh10 has been instated in France (clinicaltrials.gov NCT01801709) [283] and in the United States [285]. Therefore, CNS targeted AAV gene therapy vectors have become a promising modality for treating leukodystrophies.

- Cellular Immunodeficiencies

Nowadays, more than a hundred of primary immunodeficiency syndromes have been described. These disorders involve one or more components of the immune system, including T, B, and natural killer lymphocytes; phagocytic cells; and complement proteins [289].

The Wiskott-Aldrich syndrome (WAS) is an X-linked recessive syndrome characterized by eczema, thrombocytopenic purpura with normal-appearing megakaryocytes but small defective platelets, and undue susceptibility to infection [290]. For many years, the only potential curative therapy has been allogeneic HSC transplantation (HSCT), but HLA-mismatched HSCT may still be accompanied by unacceptable risk in some cases [291]. Gene therapy for WAS was first attempted using a retroviral vector. This approach resulted in a sustained increase in the proportion of WAS-corrected cells in all patients. However, the majority of patients developed acute leukemia secondary to viral enhancer-mediated insertional mutagenesis [292]. Recently, gene therapy approaches using a self-inactivating lentiviral vector has shown promising results in children and young adults, demonstrating the feasibility of the use of new gene therapies in WAS patients [293]. 

Severe combined immunodeficiency (SCID) consists of a group of fatal genetic disorders characterized by profound deficiencies of T- and B-cell (and sometimes NK-cell) function. Infants with SCID are lymphopenic [294]. Twenty-five years ago, bone marrow cells genetically corrected with a retroviral vector were administered to a child suffering from adenosine deaminase-deficient severe combined immunodeficiency (ADA-SCID). This was the first approach to using genetically modified stem cells to treat a human disease [295]. In 2016, the pharmaceutical company GlaxoSmithKline announced that the European Medicines Agency had granted marketing authorization to Strimvelis, the commercial name of gene therapy for ADA-SCID [296].

- Neuronal Neuropathies

Giant axonal neuropathy (GAN) is a rare childhood onset autosomal recessive neurodegenerative disorder affecting the central and peripheral nervous system [297]. Mutations in the GAN gene cause loss of function of gigaxonin, a cytoskeletal regulatory protein, clinically leading to progressive sensorimotor neuropathy, reduced coordination, slurred speech, seizures, and progressive respiratory failure leading to death [298]. Recently, AAV9-mediated GAN gene transfer has been shown as a potential therapeutic approach for improved patient outcomes in GAN, a study which is currently being conducted as a Phase I trial at the National Institutes of Health Clinical Center (clinicaltrials.gov identifier: NCT02362438) [299,300].

- Respiratory Ciliopathies

Ciliopathies are a growing class of disorders caused by abnormal ciliary axonemal structure and function [301]. Current treatment of ciliopathies is limited to symptomatic therapy. However, the growing understanding of ciliopathy genetics coupled with recent advances in gene delivery and endogenous gene and transcript repair, demonstrated thus far in tissues of the eye, nose, and airway, offers hope for curative measures in the near future [301]. 

Primary ciliary dyskinesia (PCD) is a rare childhood disease, the prototype for motile ciliary dysfunction. PCD results from abnormal ciliary function, leading to neonatal respiratory distress, chronic sinopulmonary disease causing sinusitis, bronchiectasis, recurrent ear infections, and infertility. The goal for the management of PCD is to prevent exacerbations and complications as much as possible, and to slow the progression of disease [302].

A PCD-like phenotype caused by reduced generation of multiple motile cilia (RGMC) has been described, which is characterized by sinopulmonary symptoms and fertility defects similar to those observed in PCD patients [303,304]. The residual motile cilia in RGMC caused by mutations in CCNO (encoding cyclin O) show a normal ciliary beat, while the few remaining motile cilia in patients with RGMC caused by MCIDAS mutations are immotile. However, no situs defects have been observed in any patients with RGMC, thus suggesting that the function of nodal cilia is intact [305].

Current gene therapy still has many disadvantages, such as the lack of safe and effective methods to inject a permanently active gene, which further prevents its further repetitions in terms of multiple applications in genetic diseases. Besides, in some clinical trials, patients showed undesired immune responses to the treatment and decreased therapeutic effect over time. Finally, new gene-delivery vectors remain to be discovered and developed with better efficiency and safety than current viral vectors, opening the door for biopolymer-based non-viral approaches as safer and alternative to the aforementioned ones.

## 9. Conclusions

Our current knowledge on the use of chitosans as non-viral gene delivery vectors has still important gaps. The role of chitosan’s *M*_w_ and DA on the various physicochemical and biological phenomena associated to gene transfection has been addressed in many studies, and one undisputable conclusion is that there is not “one-size-fits-all” chitosan. More systematic approaches are necessary, particularly aimed to expand our understanding on the influence of chitosan’s structure on pDNA and siRNA molecular conformation, size, shape, and surface characteristics of complexes obtained either by direct electrostatic polyelectrolyte complexation, by co-crosslinking by ionic gelation (e.g., with TPP) or covalently (e.g., with genipin), co-complexed with other polyanions (e.g., hyaluronan, alginate) or with proteins or phospholipids. This knowledge is essential to aid in the rational design of virus-like particles with tailor made characteristics. 

Thus far, the results accrued from techniques such as SPR, fluorescence spectroscopy and ITC to probe the interaction between chitosans and/or miRNA have been instrumental to allow measuring quantitatively the binding affinity constants of chitosans of varying *M*_w_ and DA. The evidence seems consistent with the notion that there is a narrow window of affinity for a given chitosan–nucleic acid pair, where the transfection efficiency is maximized. Although the reason behind this is not fully understood, it seems that this is due to a compromise between a binding affinity which needs being high enough to condense and protect the integrity of the gene, but, at the same time, weak enough to allow the intracellular dissociation and unpacking needed to release the gene in its functional form. Whether the nature of the molecular interactions at play is merely non-specific electrostatic or of other type is unknown. It is also not known whether specific pattern sequences of acetylated and deacetylated sugar units in chitosan have specific binding affinity for given gene sequences in DNA or RNA in each of their preferred conformations. Theoretical and computational chemistry approaches are urgently needed in these regards. Gleaning this intelligence will surely open huge possibilities for specific partially acetylated chitosan oligomers, for example, to develop riboswitches, novel ribozymes or as specific therapeutic gene targets. The ongoing revolution brought about by the recent discovery of CRISPR-Cas9 technology for surgical scar-less genome editing will undoubtedly be a game changer in this field on the short term. In the field of rare diseases, gene therapy is perhaps where the greatest potential lies at first, before the costs entailed in this technology start to decrease and it becomes amenable for the treatment of more widespread diseases. We anticipate that specific chitosans will be key players in the translation of research to the clinic. The low cytotoxicity, low biopersistance and mucoadhesive properties of chitosans are hardly shared by any other biopolymer. Chitosan chemical derivatives have lain beyond the scope of this review; however, they surely also hold enormous promise yet to be discovered and realized in future. 

## Figures and Tables

**Table 1:** Summary of chitosans used as non-viral delivery nanovehicles to associate different types of genes.

Chitosans			Nucleic Acid Name	In Vitro Studies	Major Findings	Reference
Origin	DA (%)	*M*_w_ (kDa)				
Chitosan Seacure Natural Biopolymer Inc., USA	10	102	Plasmid containing a CMV promoter	Cos-1 cells	The highest level of expression in vitro was obtained using complexes prepared at a N/P = 2 and using a chitosan of *M*_w_ 102 kDa. This finding was 250-fold lower than that observed with the control of lipofectamine. Further improvement in transfection efficiency was achieved by the presence of a pH-sensitive endosomolytic peptide in complex formulation.	[47]
	20	230				
	18	540				
	10	7–92 serie				
Chitopharm^®^ Cognis Deutschland GmbH & Co., Germany	5	8.9	siRNA-eGFP duplex	H1299	Physicochemical properties and in vitro gene silencing of chitosan/siRNA nanoparticles are strongly dependent on chitosan *M*_w_ and DA. Chitosan/siRNA formulations (N/P = 50) prepared with low *M*_w_ (~10 kDa) showed almost no knockdown of eGFP, whereas those prepared from high *M*_w_ (64.8–170 kDa) and DA = 20% showed greater gene silencing ranging between 45% and 65%. The highest gene silencing efficiency (80%) was achieved using chitosan/siRNA nanoparticles at N:P 150 using higher *M*_w_ (114 and 170 kDa) and DA = 16%.	[48]
	23	11.9				
	22	64.8				
	16	114.2				
	16	170				
	46	173				
Molecular tailored chitosans	<0.2	8	DNA pWizLuc (6.7 kb)	---	Tailored chitosans include linear (LCO), trisaccharide substituted-(TCO), and self-branched trisaccharide (SB-TCO) substituted chitosan oligomers. This study revealed that, besides differences in the stability of complexes, SB-TCO and DNA formed structures with a larger height and a larger fraction of globular structures compared to the other chitosans. In addition, complexes formed by SB-TCO contained a larger fraction of unbound chitosan, which may lead to increased transfection.	[51]
		9,8				
		21				
Piramal Healthcare, India	8	8	eGFPLuc (6.4 kb)	---	Chitosan buffering capacity and its comparison to PEI on a molar basis revealed that chitosan possesses a higher buffering capacity than PEI in the endosomal pH range. Chitosan–DNA complex alone have an ~2-fold reduced buffering capacity as compared to free chitosan. These findings suggest that the proton sponge effect could be at least partially responsible for mediating chitosan endosomal escape.	[62]
Protasan Ultrapure, Pronova Biomedical	14	270	siRNA targeting pGL3 luciferase	CHO K1 HEK 293	Chitosan–TPP nanoparticles with entrapped siRNA are shown to be better vectors as siRNA delivery vehicles compared to chitosan–siRNA complexes possibly due to their high binding capacity and loading efficiency.	[67]
	14	210				
Sigma-Aldrich	1	5	DNA	--	Chitosan (5 kDa)/DNA complexes remain in B conformation, and the binding affinity of chitosan to DNA is dependent on pH of media where a great binding affinity is generated at pH 5.4, whereas at pH 12.0 a low affinity with DNA is observed. The charge ratios of chitosan to DNA strongly influence the morphology of complexes formed. At low charge ratio, not all DNA can be entrapped in the complex; at higher ratios, the complexes without free DNA evolve into spherical shape with mean size of nanoscale.	[66]
Kitomer (Marinard, Canada)	19	500	Linear calf thymus DNA (13 kbp)	---	A panel of biophysical techniques (conductivity, zeta potential, dynamic light scattering, atomic force microscopy, circular dichroism and UV/VIS spectroscopy) were used to determine the stoichiometry, net charge, dimensions, conformation and thermal stability of complexes of varying N/P ratio both in water and in 10 mM NaCl. Complexation of partially denaturated DNA in water, and double-helical DNA, showed similar electrostatic behavior and stoichiometry. The behavior for complexing was nearly independent of *M*_w_.	[101]
Primex	4	50				
Protasan^®^ UP G 113, Sigma-Aldrich	10–25	160	miR126	CFBE41o−	High-content analysis data indicate that miRNA-PEI nanomedicines facilitated greater uptake than miRNA-TPP-chitosan nanoparticles and the commercial transfection agent, RiboJuice^®^. Polymeric nanoparticles can deliver premiRs effectively to CFBEs to modulate gene expression but must be tailored specifically for miRNA delivery.	[71]
Chitosan Aldrich Chemical Co.	12	213	pEGFP-C2 plasmid	A549	DNA condensation of N90% was achieved at the N/P charge ratio of 6, independent of the chitosan *M*_w_ and DA. NP produced with chitosan of *M*_w_ 213 kDa and DA of 12% showed the highest zeta potential (+23 mV), cellular uptake (4.1 Ag/mg protein) and transfection efficiency (12.1%), while chitosan vector with *M*_w_ of 213 kDa and DA of 54% showed the lowest cellular uptake (0.4 Ag/mg protein) and transfection efficiency (0.05%)	[73]
	12	98				
	12	48				
	12	17				
	12	10				
	39	213				
	54	213				
KITTOLIFE, Korea.	27.5	22	pSV-β-galactosi-dase	293T	The transfection efficiency of low *M*_w_ chitosan complexes (LMWC) was significantly higher than naked DNA and higher than poly-Lysine (PLL). MTT assay showed that LMWC was less cytotoxic than PLL.	[74]
Seafresh Chitosan Lab, Thailand	13	20	pcDNA3-CMV-Luc	CHO-K1	The transfection efficiency of chitosan (CS)/DNA complexes was dependent on the salt form and *M*_w_ of chitosan, and the N/P ratio of CS/DNA complexes. Of different CS, the maximum transfection efficiency was found in different N/P ratios. CS/DNA (hydrochloric acid), CS/DNA (lactic acid), CS/DNA (acetic acid), CS/DNA (aspartic acid) and CS/DNA (d glutamic acid) complexes showed maximum transfection efficiencies at N/P ratios of 12, 12, 8, 6 and 6, respectively. Cytotoxicity results showed that all CS/DNA complexes had low cytotoxicity.	[76]
	13	45				
	13	200				
	13	460				
Chitosan Seacure Pronova Biopolymers, Norway	15	6.6	pcDNA3-luc	EPC cells	The in vitro transfection efficiency was affected by the polyplex (N/P) charge ratio, the DNA concentration in the complexes, the molecular weight and degree of acetylation of the chitosans. Two favorable formulations were identified: chitosan (DA-15%; 6.6 kDa) (theoretical charge ratio 10) and chitosan (DA-15%; 160 kDa) (theoretical charge ratio 3). The size of the complexes was affected by the degree of acetylation, concentration of DNA, pH, inclusion of a coacervation agent and the charge ratio.	[75]
	32	90				
	15	160				
	25	160				
Biosyntech, Laval, Canada	20	40	eGFPLuc	HEK293	The kinetics of decondensation in relation to lysosomal escape was a most critical structure-dependent process affecting the transfection efficiency of chitosan polyplexes. The most efficient chitosans showed an intermediate stability and a kinetics of dissociation, which occurred in synchrony with lysosomal escape. In contrast, a rapid dissociation before lysosomal escape was found for the inefficient high DA chitosan whereas the highly stable and inefficient complex formed by a high *M*_w_ and low DA chitosan did not dissociate even after 24 h.	[77]
	8	10				
	8	150				
	28	40				
Molecular tailored chitosans	all 0.2	146	gWiz Luc and gWiz GFP	HEK293	Maximum level of transgene expression was found with chitosan with *M*_w_ 8 and 11.6 kDa. An increase in chain length and/or the amino-phosphate (A/P) ratio reduced and delayed transgene expression. The gene transfer pattern correlated with the ability of heparin to release DNA from the polyplexes. As a tool to facilitate the unpacking of the polyplexes, we substituted the chitosans with uncharged oligosaccharides that reduced the interaction with DNA. The substitution of chitosans shorter than 4.7 kDa completely abolished transfection.	[78]
		32.9				
		24.8				
		16.4				
		11.6				
		8.0				
		4.7				
Sascha Mahtani Chitosan PVT Ltd., India	1.5	26	miRNA-145	MCF-7	Chitosan–miRNA nanocomplexes with degree of acetylation 12% and 29% were biologically active, showing successful downregulation of target mRNA expression in MCF-7 cells. We found no evidence that these complexes were cytotoxic towards MCF-7 cells. DA has an influence on the transfection efficiency for complexes with equivalent (+/−) charge ratio (8.0): more efficient downregulation of the target gene in the presence of intermediate DA (~30%)	[81]
	12	25				
	29	20				
	49	18				
	1.6	1.3				
	11	1.2				
	25	1.1				
	67	1.9				
Sigma-Aldrich	15–25	192	siRNAs targeting the VEGF gene	DLD-1	Particles with different cross-linkers were prepared. Chitosan–TPP nanoparticles showed better siRNA protection during storage at 4 °C. TEM micrographs revealed the assorted morphology of chitosan–TPP–siRNA nanoparticles in contrast to irregular morphology displayed by chitosan–DS–siRNA and chitosan–PGA–siRNA nanoparticles. All siRNA loaded chitosan–TPP–DS–PGA nanoparticles showed initial burst release followed by sustained release of siRNA. All the formulations showed low and concentration-dependent cytotoxicity with human colorectal cancer cells (DLD-1), in vitro. The cellular uptake studies with chitosan–TPP–siRNA nanoparticles showed successful delivery of siRNA within cytoplasm of DLD-1 cells.	[84]
Sigma-Aldrich	--	Low	pEGFPN1	HEK293	Suitable candidate for gene delivery would be alginate–chitosan nanoparticles. The effect of alginate on reducing the strength of electrostatic interactions between chitosan and pDNA, resulting in better transfection and increasing the plasmid release.	[86]
Protasan UP CL 113 FMC Biopolymers (Norway)	10–15	~110	pEGFP-C1 and pβ-gal	HCE; IOBA-NHC	Evidence of the potential of hyaluronic acid (HA)–chitosan nanoparticles, which exhibit very low cytotoxicity for the targeting and further transfer of genes to the ocular surface.	[87]
Ditto	10–15	~110	pSEAP	IOBA-NHC; HCE; RAW264.7	HA-chitosan oligomer (CSO)-based nanoparticles (HA–CSO NPs) were internalized by two different ocular surface cell lines by an active transport mechanism. Potential use of HA–CSO NPs to deliver genetic material to the ocular surface.	[88]
Ditto	10–15	~110	Luciferase duplex siRNA (21 bp)	A549-Luc	Chitosan–TPP nanoparticles without and with HA (CS–TPP–siRNA and CS–TPP–HA–siRNA, respectively); N/P charge ratio 5–200, diameter ~320–420 nm. Inclusion of HA reduced the cytotoxicity. Greater inhibition of luciferase expression was for CS–HA NPs (N/P = 120)-luciferase knockdown of ~85% (vs. <70% for CS–TPP–siRNA).	[102]
Ditto	10–15	~110 ~10 (depolyme-rized)	gWiz^TM^ pSEAP	Calu-3 (in air-liquid interface)	Chitosan–TPP NPs comprising anionic β-cyclodextrins; 5% DNA loading; diameter ~264–358 nm. Slightly lower cytotoxicity for NPs comprising CDs; interaction of NPs with Calu-3 cells studied by CLSM. Pharmacokinetics of SEAP expression: NPs of chitosan–TPP, and chitosan–TPP–carboxymethyl-β-CD had greater transfection efficiency than those comprising sulfobutylether-β-CD. In NPs comprising chitosan of ~10 kDa, this effect was not observed.	[103]
Fluka	--	111	pCMV Lac-Z (7kbp)	HeLa	Chitosan–DNA polyplexes of N/P charge ratio 1–20. The chitosan of higher valence (*M*_w_) required larger amounts to compact DNA. A 4–5-fold lower enthalpic contribution observed for the highest valence chitosan. Heterogeneous population of particles in the diameter range ~250–500 nm. Very low transfection efficiency observed for all systems, ascribed to the core-shell morphology of the polyplexes.	[104]
	--	266	pCMV-Luc			
	--	467	(6kbp)			
Shrimp shell	24	--	--	L929 BHK21 (C13)	Biocompatibility was investigated for: (1) cell adherence and growth on the chitosan samples as substrate; (2) the effect of extract media on 2-day and 7-day growth; and (3) the presence of an inhibition zone. The results were similar for both cell lines.	[89]
	14	--				
Cuttlefish	19	--				
	10	--				
UltrasanTM, Biosyntech Inc., Canada	2	120	eGFPLuc	HEK293	Results revealed an important coupling between DA and *M*_w_ of chitosan in determining transgene expression. Maximum expression was obtained with a certain combination of DA and *M*_w_ that depended on N/P charge ratio and the pH.	[90]
	8	200				
	20	320				
	28	220				
Vanson, USA	10	390	pcDNA encoding for Luc.	HEK293 HeLa SW756	Degree of chitosan deacetylation is an important factor in chitosan–DNA nanoparticle formulation as it affects DNA binding, release and gene transfection efficiency in vitro and in vivo.	[91]
	30	209				
	38	138				
Marinard, Canada	28	10	siRNA	EGFP + H1299	Highly deacetylated chitosans are superior siRNA delivery systems compared to partially acetylated chitosans. Highly deacetylated chitosans (low DA and high *M*_w_) provide the optimal balance between biological performance and toxicity. A minimum polymer length of ~60−70 monomers (~10 kDa) was required for stability and knockdown. In vitro knockdown was equivalent to lipid control with no metabolic or genotoxicity. An inhibitory effect of serum on biological performance was dependent on DA, *M*_w_, and N/P charge ratio. In vivo biodistribution in mice show accumulation of nanoparticles in kidney with 40–50% functional knockdown.	[93]
		120				
	20	10				
		120				
	8	5				
		10				
		40				
		80				
		120				
	2	10				
		120				
Yaizu Industry, Japan	20	15	pGL3	A549 HeLa B16	Transfection efficiencies of the pGL3/chitosan complexes were dependent on pH of culture medium, stoichiometry of pGL3: chitosan, serum, and molecular mass of chitosan.	[94]
	6	52				
	8	>100				
Sigma–Aldrich	34	50	pMAX-eGFP	HEK293 H441 16HBE	The morphology and the net charge of chitosan–pDNA aggregates is mainly controlled by the overall stoichiometric ratio between the positively charged (protonated) groups on chitosan chains and the negative charges on the DNA. Complexes with the higher molecular weight chitosan are more stable, and clearly demonstrate a significantly higher transfection efficiency.	[95]
	43	150				
KiOmedicine-CsU from *Agaricus bisporus* (3–5% glucan content)	14	44	siRNAluc GI3	H1299 pGL3 (expressing Luc reporter gene)	Comparisons of biophysical and transfection efficiency properties of fungal chitosans with similar DA, and *M*_w_ from 44kDa to 143 kDa (N/P ~4 and 8). Polyplexes despite very similar size (129–165 nm), zeta potential (+20–30 mV) and complex stability *(K*_d_ = 1–1.9 nM), displayed differences in particle morphology, cellular uptake and transfection efficiency. Spherical or elongated and irregular nanoparticles were formed with chitosans of *M*_w_ 44 (DA 14%) or 143 kDa (DA 22%), respectively. First study to use ITC for profiling of siRNA interactions with chitosan.	[98]
	16	63				
	19	93				
	22	144				
Protasan UPG Pronova Biopolymer, Norway	0.1	1.2–10	gWizTM-Luc pCMV-Luc	HEK293 HeLa	A major improvement of chitosan-mediated non-viral gene delivery to the lung was obtained by using polyplexes of well-defined chitosan oligomers. Polyplexes of oligomer fractions also had superior physicochemical properties to commonly used high-molecular-weight ultrapure chitosan (UPC).	[100]
Norwegian Biopolymer Laboratory	0	5–6	pNGVL-eGFPLuc	CFBE41o− HEK293	The transfection efficacy of polyplexes in the CFBE41o− cell line was poor compared with that in HEK293 cells. The narrow-size-distributed chitosan at low pH shows a better transfection efficiency compared with PEI.	[105]
HMC+, Germany	30	20	pEGFP-C1 or pEGFP-C1/siRNA	CFBE41o−	Proof-of-principle that co-transfection with chitosan, as a natural non-toxic vector, might be an effective delivery system in a human CF cell line, reaching comparable levels to those achieved using lipid-based systems.	[106]
HMC+, Germany	30	20	wtCFTR-mRNA	CFBE41o−	Transfection of an immortalized CF cell line with wtCFTR-mRNA using chitosan as a carrier results in increased CFTR function	[107]
HMC+, Germany	30	30	CFTR-LNA	--	CFTR-specific locked nucleic acids (LNA) biopolymer-based nanoparticles represent a promising system for further development of new lung-targeted CF therapeutic approaches. First time the use of chitosan from a non-animal source as a potential therapeutic vector has been reported.	[108]
ChiPro, Germany	20	200				

## Abbreviations

AADC	Aromatic l-amino acid decarboxylase
AAV	Adeno-associated virus
ADA-SCID	Adenosine deaminase-deficient severe combined immunodeficiency
AFM	Atomic force microscopy
ARSA	Deficiency of lysosomal arylsulfatase A
ASOs	Antisense oligonucleotides
cAMP	Cyclic adenosine monophosphate
CD	Circular dichroism
cDNA	Complementary deoxyribonucleic acid
CF	Cystic fibrosis
CFTR	Cystic fibrosis transmembrane conductance regulator
CLSM	Confocal laser scanning microscopy
CMV	Cytomegalovirus
CNS	Central nervous system
CPP	Cell penetrating peptide
CRISPR	Clustered regularly interspaced short palindromic repeats
CS	Chitosan
DA	Degree of acetylation
DHM	Digital holographic microscopy
DLS	Dynamic light scattering
DP	Degree of polymerization
DS	Dextran sulphate
dsRNA	Double-stranded ribonucleic acid
eGFP	Green fluorescent protein
ENaC	Epithelial sodium channel
GAN	Giant axonal neuropathy
HA	Hyaluronic acid
HDP	High degree of polymerization
HSC	Hematopoietic stem cells
ITC	Isothermal titration calorimetry
LDV	Laser Doppler velocimetry
LNA	Locked nucleic acids
LPLD	Familial lipoprotein lipase deficiency
LSD	Lysosomal storage diseases
miRNA	Micro ribonucleic acid
MLD	Methachromatic leukodistrophy
mRNA	Messenger ribonucleic acid
*M*_w_	Molar mass
N/P	Amino to phosphate charge ratio
NPs	Nanoparticles
oc pDNA	Open chain plasmid deoxyribonucleic acid
PA	Pattern of acetylation
PAMPs	Pathogen-associated molecular patterns
PCD	Primary ciliary dyskinesia
PDE	Phosphodiesterase
pDNA	Plasmid deoxyribonucleic acid
PEI	Polyethylene imine
PGA	Poly-d-glutamic acid
PLL	Poly-Lysine
rAAV	Recombinant adeno-associated virus
rAD	Recombinant adenovirus
RGMC	Reduced generation of multiple motile cilia
RISC	RNA interference silencing complex
rLV	Recombinant lentivirus
RT-PCR	Real-time quantitative reverse transcription polymerase chain reaction
SAXS	Small angle X-ray scattering
SCID	Severe combined immunodeficiency
SeV	Murine parainfluenza virus type 1
siRNA	Small interfering ribonucleic acid
sc pDNA	Supercoiled plasmid deoxyribonucleic acid
SPR	Surface plasmon resonance
ssRNA	Single-stranded ribonucleic acid
TALEN	Transcription activator-like effector nucleases
TEM	Transmission electron microscopy
TLR	Toll-like receptor
TPP	Pentasodium tripolyphosphate
WAS	Wiskott-Aldrich syndrome
ZFN	Zinc finger nucleases
